# MinION sequencing technology to characterize unauthorized GM petunia plants circulating on the European Union market

**DOI:** 10.1038/s41598-019-43463-5

**Published:** 2019-05-09

**Authors:** Marie-Alice Fraiture, Gabriella Ujhelyi, Jaroslava Ovesná, Dirk Van Geel, Sigrid De Keersmaecker, Assia Saltykova, Nina Papazova, Nancy H. C. Roosens

**Affiliations:** 1 Sciensano, Transversal activities in Applied Genomics (TAG), J. Wytsmanstraat 14, 1050 Brussels, Belgium; 2 National Food Chain Safety Office, Mester utca 81, H-1024 Budapest, Hungary; 3 Crop Research Institute, Department of Molecular Genetics, Drnovská 507, 161 06 Prague, Czech Republic

**Keywords:** Molecular engineering in plants, Next-generation sequencing

## Abstract

In order to characterize unauthorized genetically modified petunia, an integrated strategy has been applied here on several suspected petunia samples from the European market. More precisely, DNA fragments of interest were produced by DNA walking anchored on key targets, earlier detected by real-time PCR screening analysis, to be subsequently sequenced using the MinION platform from Oxford Nanopore Technologies. This way, the presence of genetically modified petunia was demonstrated via the characterization of their transgene flanking regions as well as unnatural associations of elements from their transgenic cassette.

## Introduction

Recently, genetically modified (GM) petunia plants, imported from firms located in Germany and the Netherlands, have been found on the Finnish market by the Finnish Food Safety Authority^1^. Afterwards, GM petunia plants have also been detected in other EU countries. Regarding the environment as well as the food and feed chain, GM petunia plants have been assessed by experts as representing a negligible risk. However, the presence of GM petunia plants on the European (EU) market is considered as illegal because no authorization has been delivered by the EU competent authorities regarding their cultivation and commercialization. Consequently, all GM petunia plants identified on the EU market have to be discarded and destroyed. However, given that no registration of commercial names for petunia varieties is currently mandatory, the potential identification of GM petunia events based on commercial names is complicated by the fact that a single petunia variety may have several different commercial names and *vice versa*^1–5^.

To screen the potential presence of GM organisms (GMO) in the food and feed chain, the enforcement laboratories are usually using real-time PCR methods targeting transgenic elements commonly found in GMO. Subsequently, GM events are identified using the corresponding real-time PCR event-specific method^6^. In the context of GM petunia, the same screening strategy can be employed in targeting transgenic elements previously used in the generation of GM petunia, such as p35S (Cauliflower mosaic virus (CaMV) 35S promoter), t35S (CaMV 35S terminator), pNOS (*Agrobacterium tumefaciens* nopaline synthase promoter), tNOS (*Agrobacterium tumefaciens* nopaline synthase terminator), nptII (neomycin phosphotransferase II gene) and tOCS (*Agrobacterium tumefaciens* octopine synthase terminator). In addition, GM petunia plants harboring the maize *A1* gene (dihydroflavonol 4-reductase), allowing to modify the flower pigmentation, have previously been reported. Therefore, a screening marker targeting this maize *A1* gene can also be interesting to be used^3–5,7–16^. Although it is necessary for the identification as well as to trace the origin, no real-time PCR construct-specific method and event-specific method is available for GM petunia. Moreover, the development of real-time PCR construct-specific and event-specific methods requires first of all an access to their genome or at least to crucial sequences such as their transgene flanking regions and unnatural associations of elements from their transgenic cassette(s). Moreover, given that the GM petunia plants may have different origins of transformation, more than one method could be necessary to develop.

With the aim to get the sequences of interests corresponding to transgene flanking regions and unnatural associations of transgenic elements, we have applied on petunia samples an integrated strategy previously developed to detect and demonstrate the presence of unauthorized GMO in the food and feed chain^17–24^. This strategy consists of two main successive steps. First, the presence of GMO is detected through a real-time PCR screening targeting key transgenic elements potentially commonly found in GMO, especially in GM petunia. Second, a PCR-based DNA walking method anchored on earlier detected key transgenic elements is applied bi-directionally to obtain information about the sequences flanking the known transgenic elements via the sequencing of the resulting PCR products using a long-length Next Generation Sequencing (NGS) technology, which was in this study the MinION platform from Oxford Nanopore Technologies. In order to detect and trace GM petunia plants, the proposed integrated strategy has thus been in this study applied on several petunia plants commercialized on the EU market.

## Results and Discussion

**Figure 1:**
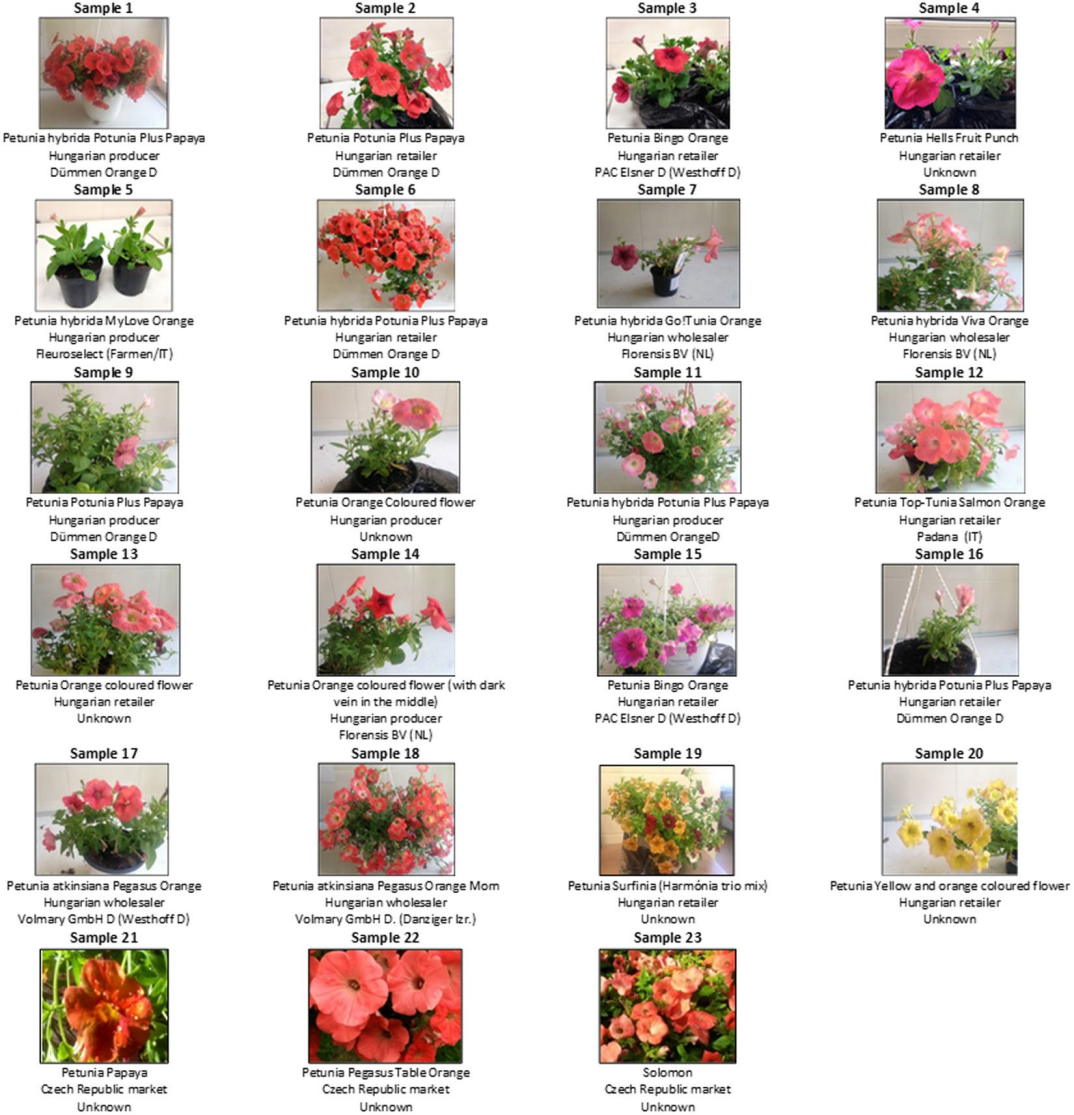
List of petunia samples. For each sample, the commercial name, origin and distributor are indicated in that order below the corresponding picture.

Several petunia plants commercialized on the EU market were collected in Hungary and the Czech Republic (Fig. 1). First, the petunia samples were screened for the potential presence of GM petunia by real-time PCR screening using markers targeting elements that are either commonly found in GMO (p35S, pNOS, tNOS, Cry1Ab/Ac and t35S) or only present in EU unauthorized GMO (t35S pCAMBIA). The screening marker targeting the maize *A1* gene, was also integrated in the analysis given its reported presence in GM petunia plants. An additional category of screening markers specific to the *Cauliflower Mosaic Virus* (CaMV) (CAP and CRT2) was used to discard false positive signals coming from transgenic sequences derived from CaMV, such as p35S (Table 1)^4,5,17–24^. As expected, the wild-type (WT) petunia plants (samples n°19–20) were negative for all tested screening markers. All other samples (n°1–18 and n°21–23) presented a similar pattern of real-time PCR amplification. These latter presented a positive signal for the p35S, pNOS, t35S and A1 screening markers and a negative signal for the tNOS, Cry1Ab/Ac, t35S pCAMBIA, CAP and CRT2 screening markers (Table 2).

**Table 1:** Oligonucleotides used for the real-time PCR screening, DNA walking and PCR assays.

Assays	Methods	Oligonucleotide names	Oligonucleotide sequences	References
Real-time PCR screening	p35S	p35S-F	AAAGCAAGTGGATTGATGTGATA	^27^
		p35S-R	GGGTCTTGCGAAGGATAGTG	^27^
Real-time PCR screening	pNOS	pNOS-F	CGTTTTACGTTTGGAACTGACA	^28^
		pNOS-R	CTCATTAAACTCCAGAAACCCG	^28^
Real-time PCR screening	tNOS	tNOS-F	GATTAGAGTCCCGCAATTATACATTTAA	^27^
		tNOS-R	TTATCCTAGKTTGCGCGCTATATTT	^27^
Real-time PCR screening	t35S pCAMBIA	t35S pCAMBIA-F	CGGGGGATCTGGATTTTAGTA	^17^
		t35S pCAMBIA-R	AGGGTTCCTATAGGGTTTCGCTC	^17^
Real-time PCR screening	Cry1Ab/Ac	Cry1Ab/Ac-F	ACCGGTTACACTCCCATCGA	^29^
		Cry1Ab/Ac-R	CAGCACCTGGCACGAACTC	^29^
Real-time PCR screening	t35S	t35S-F	In-house	Unpublished data
		t35S-R	In-house	Unpublished data
Real-time PCR screening	CAP_CaMV	CAP-F	In-house	Unpublished data
		CAP-R	In-house	Unpublished data
Real-time PCR screening	CRT2_CaMV	CRT2-F	CCAGAAGAACATTGGGTCAATGC	^30^
		CRT2-R	CTATGTCTTTGCAGACTTTGCTGAT	^30^
Real-time PCR screening	A1	qPhA1_F4	CGACTTCTGCCGTCGCG	^5^
		qPhA1_R4	GATGATGGTGACCAGGTCCAG	^5^
DNA Walking	p35S-F	p35S-F a	GGGTCTTGCGAAGGATAGTG	^27^
		p35S-F b	TGTGCGTCATCCCTTACGTCAGT	^19^
		p35S-F c	TATCACATCAATCCACTTGCTTT	^27^
DNA Walking	p35S-R	p35S-R a	AAAGCAAGTGGATTGATGTGATA	^27^
		p35S-R b	ACTGACGTAAGGGATGACGCACA	^19^
		p35S-R c	CACTATCCTTCGCAAGACCC	^27^
PCR confirmation assay	GM petunia transgene flanking region	Junction-F	CATTTCGCCCTCATGAAAATGAT	This study
		Junction-R	GGTCGCCGCATACACTATTC	This study

**Table 2:** Results of the real-time PCR screening analysis, using the p35S, pNOS, tNOS, t35S pCAMBIA, Cry1Ab/Ac, t35S, A1, CAP and CRT markers, and the PCR confirmation assay, targeting the GM petunia transgene flanking region (junction), applied on the petunia samples.

Samples	Real-time PCR screening analysis									PCR confirmation assay
	p35S	pNOS	tNOS	t35S pCAMBIA	Cry1Ab/Ac	t35S	CAP	CRT2	A1	Junction
1_GM	+	+	−	−	−	+	−	−	+	+
2_GM	+	+	−	−	−	+	−	−	+	+
3_GM	+	+	−	−	−	+	−	−	+	+
4_GM	+	+	−	−	−	+	−	−	+	+
5_GM	+	+	−	−	−	+	−	−	+	+
6_GM	+	+	−	−	−	+	−	−	+	+
7_GM	+	+	−	−	−	+	−	−	+	+
8_GM	+	+	−	−	−	+	−	−	+	+
9_GM	+	+	−	−	−	+	−	−	+	+
10_GM	+	+	−	−	−	+	−	−	+	+
11_GM	+	+	−	−	−	+	−	−	+	+
12_GM	+	+	−	−	−	+	−	−	+	+
13_GM	+	+	−	−	−	+	−	−	+	+
14_GM	+	+	−	−	−	+	−	−	+	+
15_GM	+	+	−	−	−	+	−	−	+	+
16_GM	+	+	−	−	−	+	−	−	+	+
17_GM	+	+	−	−	−	+	−	−	+	+
18_GM	+	+	−	−	−	+	−	−	+	+
21_GM	+	+	−	−	−	+	−	−	+	+
22_GM	+	+	−	−	−	+	−	−	+	+
23_GM	+	+	−	−	−	+	−	−	+	+
19_WT	−	−	−	−	−	−	−	−	−	−
20_WT	−	−	−	−	−	−	−	−	−	−

Positive and negative signals are respectively indicated by + and −. The samples are indicated as genetically modified (GM) or wild-type (WT).

In a second step, the samples suspected to be GM petunia (n°1–18 and n°21–23) were analyzed with the available bidirectional DNA walking method anchored on the p35S transgenic element that presented a positive signal during the previous real-time PCR screening step^19,21,24^. All the generated PCR products presented a size range going approximately from 100 to 1500 bp. The obtained PCR products of each petunia sample were pooled and barcoded to be then sequenced using the MinION platform from Oxford Nanopore Technologies (Supplementary File 1).

**Figure 2:**
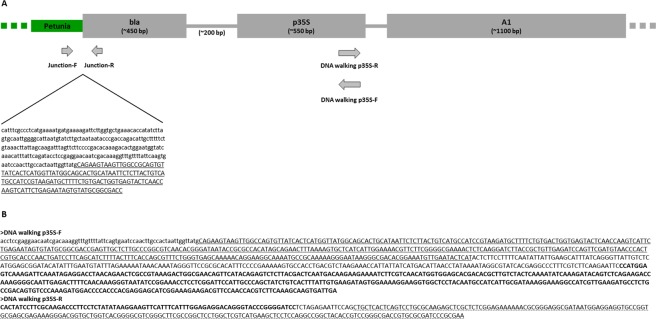
Schematic representation of the identified transgenic cassette of the tested GM petunia plants, based on the sequence information generated in this study. (**A**) The petunia part is represented in green while the transgenic cassette is indicated in grey. The approximate size of the transgenic elements is indicated in base-pairs (bp). The beta-lactamase gene (*bla*); the Cauliflower Mosaic Virus (CaMV) 35S promoter (p35S); the maize *A1* gene (*A1*). The starting positions and walking directions of the applied DNA walking methods anchored on the p35S element are indicated by arrows (Table 1). The positions of primers (Junction-F and Junction-R) used to confirm the observed transgene flanking region with the MinION platform are indicated by arrows (Table 1). The consensus sequences of the transgene flanking region confirmed by PCR amplification and Sanger sequencing for the petunia samples n°1–18, 21–23 (see Supplementary File 5) is indicated. (**B**) The consensus sequences produced by the MinION platform with the DNA walking p35S-F (see Supplementary Files 3–4) and the DNA walking p35S-R (see Supplementary File 2) methods applied on all GM petunia samples are indicated. In all these sequences, the petunia part is indicated in lower case while the transgenic cassette, containing *bla* (underlined), uncharacterized vector part, p35S (in bold) and the maize *A1* gene (dotted underlined), is indicated in upper case.

Among the generated DNA fragments from each tested petunia sample, the ones related to the DNA walking p35S-R method were all characterized by a sequence successively corresponding to the p35S element [GenBank: MF521566.1; AY615305.1] followed by an uncharacterized vector sequence [GenBank: MF521566.1] and the maize *A1* gene [GenBank: MF521566.1; NM_001158995.2]. This association of sequences was previously mentioned as being used to produce GM petunia presenting a novel flower pigmentation^7^. Regarding the sequences of the DNA fragments produced with the DNA walking p35S-F method, the p35S element [GenBank: KY964325.1] was followed by an uncharacterized vector sequence [GenBank: KY964325.1] and the beta-lactamase (*bla*) gene [GenBank: KY964325.1; KT405500.1], conferring a resistance to ampicillin (Fig. 2, Supplementary Files 2–4). The identification of these unnatural associations of elements allows confirming that these petunia plants were actually GM. These results suggest also that there is the possibility that the same transgenic cassette may have been used in all the GM petunia tested in this study. Moreover, these sequences could be useful to develop a construct-specific method. For most of the petunia samples (n°1–3, n°5, n°7–9, n°11–14, n°16–18 and n°21–23), longest sequences were generated by the DNA walking p35S-F method, allowing to reach the transgene flanking region between the *bla* element from the transgenic cassette [GenBank: KY964325.1; KT405500.1] and the petunia host genome [GenBank: AY136628.2; CP023763.1; HG975447.1] (Fig. 2, Supplementary Files 3, 5).

As the identified transgene flanking region was highly similar between all these samples (n°1–3, n°5, n°7–9, n°11–14, n°16–18 and n°21–23), the same origin of transformation was suspected (Supplementary File 6). The confirmation of this transgene flanking region was obtained by PCR amplifications followed by Sanger sequencing using two primers, one designed on the petunia sequence and one designed on the *bla* gene (Fig. 2, Table 1, Supplementary File 6). Due to the sequence similarity between the p35S and *bla* elements for all tested GM petunia samples (Supplementary File 4), this analysis was also applied on GM petunia samples presenting shorter sequences characterizing only the transgenic cassette (n°4, n°6, n°10 and n°15) (Fig. 2, Supplementary File 3). All these GM petunia samples presented a PCR amplicon with the expected size (402 bp) and with the same transgene flanking region (Table 2, Fig. 2, Supplementary File 5). The identical insertion site of these samples suggests a similar origin of GM event transformation.

In order to collect additional information related to the petunia sequence interrupted by the inserted transgenic cassette, further analysis were performed. This petunia sequence presented a similarity (77%) with a transposon (GenBank: RVW93168.1) (Fig. 2; Supplementary File 7). The insertion of the transgenic cassette expressing the maize *A1* gene on a transposable element, well-known to be able to influence gene expression, could explain the flower color variations observed among the tested GM petunia plants^25^. However, further analysis are needed to confirm this hypothesis.

Regarding the transgenic cassette of all tested GM petunia samples, the GM petunia described in Meyer *et al*., 1987 represents a possible origin^7^. This hypothesis is based on the similarity of the unnatural association of elements (*bla*, p35S and *A1* gene) (Fig. 2, Supplementary Files 2–4) as well as to the presence and absence of transgenic elements determined using the real-time PCR screening (positive for p35S, pNOS and t35S and negative for tNOS) (Table 2). However, the analysis of the GM petunia material published by Meyer *et al*., 1987 and its comparison to the GM petunia plants tested in this study are needed to confirm this hypothesis^7^.

At the technical level, the used MinION platform from the Oxford Nanopore technologies presents the advantage to be able to deal with heterogenic DNA libraries, to provide data in real-time of long read-lengths and to be easily available in-house due to the small size and portability of the device and the attractive price. Moreover, this high-throughput technology allows to sequence several samples simultaneously. In contrast to the Sanger sequencing platform, the use of a NGS platform, such as the MinION device, for the sequencing of DNA libraries generated through the proposed PCR-based DNA walking methods allows also to reduce the laborious aspect of the experimental procedure. More precisely, as each DNA walking PCR product presents in general several amplicons, an individual purification of each of these amplicons from an electrophoresis gel is required for their subsequent sequencing using the Sanger technology while the entire PCR product can directly be sequenced using the MinION device. However, according to previous studies, this NGS technology presents a relatively high error rate of generated raw data (~10–20%)^21,24,26^. In the present study, despite the high similarity between the sequences of the transgene flanking region generated by the MinION and Sanger platforms, the error rate was varying approximately between 1 to 22% (Supplementary File 6). Nevertheless, in contrast to a single mutation point, the detection of transgenic cassettes and transgene flanking regions from GMO as well as the determination of the transformation origin were still possible with such a kind of error rate. These generated sequences could also be used to develop real-time PCR construct-specific and event-specific methods in order to be used in GMO routine analysis by the enforcement laboratories for the survey of GM petunia plants on the market. For such purpose, the use of sequences confirmed by the Sanger sequencing technology, presenting a lower error rate than the MinION platform, is strongly advised for the oligonucleotide design of these real-time PCR construct-specific and event-specific methods^24,26^.

## Conclusion

The proposed integrated strategy, combining a PCR-based DNA walking, anchored on earlier detected key transgenic elements, with the MinION sequencing platform from Oxford Nanopore Technologies, was successfully applied on several suspected GM petunia samples circulating on the EU market. Their presence was demonstrated by the characterization of their transgene flanking regions as well as unnatural associations of elements from their transgenic cassette. Moreover, the high similarity between the transgene flanking region from all tested GM petunia samples strongly suggests their common origin of transformation although these GM petunia samples had different commercial names. Given that the tested GM petunia plants were also presenting some flower color variations among themselves (Fig. 1), any petunia plants could be relevant for such investigation^5^. In addition, with the aim to increase the characterization of sequences of interest, additional DNA walking methods anchored on other transgenic elements observed in GM petunia, such as *bar*, pNOS and t35S, could be useful to develop.

Regarding the choice of the NGS platform, the MinION device allowed in-house generation of a long-read sequencing data for several samples simultaneously in a short-time frame (maximum 48 hours), which is highly interesting because such time-frame is compatible with the workflow of enforcement laboratories. However, the bioinformatics analysis still required a certain level of expertise necessary to be developed in enforcement laboratories. Moreover, given its relative high-error rate, the re-sequencing of fragments of interest using for instance the Sanger technology is advised for further applications, such as the development of real-time PCR construct-specific and event-specific methods.

## Materials and Methods

### Plant materials, DNA extraction, DNA concentration and DNA purity

Twenty-three petunia samples were collected from the Hungarian (samples n°1–20) and Czech Republic (samples n°21–23) markets (Fig. 1). Based on the real-time PCR assay (Table 2), the petunia plants corresponding to the samples n°1–18 and n°21–23 were suspected to be GM while the petunia plants corresponding to the samples n°19 and n°20 were wild-type (WT). The commercial names of the GM petunia samples were “Petunia hybrida Potunia Plus Papaya”, “Petunia Potunia Plus Papaya”, “Petunia Bingo Orange”, “Petunia Hells Fruit Punch”, “Petunia hybrida MyLove Orange”, “Petunia hybrida Go!Tunia Orange”, “Petunia hybrida Viva Orange”, “Petunia Orange Coloured flower”, “Petunia Top-Tunia Salmon Orange”, “Petunia Orange coloured flower (with dark vein in the middle)”, “Petunia atkinsiana Pegasus Orange”, “Petunia atkinsiana Pegasus Orange Morn”, “Petunia Surfinia (Harmónia trio mix)”, “Petunia Yellow and orange coloured flower”, “Petunia Papaya”, “Petunia Pegasus Table Orange” and “Solomon” (Fig. 1). These samples were sampled by the Hungarian and Czech Republic competent authorities in the GMO control context. Leaves from each sample were grounded individually to obtain a fine homogenous powder. The subsequent DNA extraction step was performed using the DNA Wizard Clean up system for genomic DNA kit (Promega) for the samples n°1–20 and a CTAB-based procedure (ISO 21571) for the samples n°21–23 (International Standard ISO 21571, 2005). DNA concentration was measured by spectrophotometry using the Nanodrop® 2000 (ThermoFisher) and DNA purity was evaluated using the A260/A280 and A260/A230 ratios.

### Real-time PCR assay

For the real-time PCR screening, 25 ng of DNA from each sample were tested using the p35S, pNOS, tNOS, t35S pCAMBIA, Cry1Ab/Ac, t35S, A1, CAP and CRT2 markers (Table 1). The real-time PCR mix contained 1X SYBR®Green PCR Mastermix (Diagenode) and 250 nM of each primer in a total volume of 25 µl. The real-time PCR program consisted of initial DNA polymerase activation for 10 min at 95 °C followed by 40 amplification cycles of 15 sec at 95 °C (denaturing step) and 1 min at 60 °C, for the p35S, pNOS, tNOS, t35S pCAMBIA, Cry1Ab/Ac, t35S, CAP and CRT2 markers, or 65 °C, for the A1 marker, (annealing-extension step). Melting curve analyses were performed by gradually increasing the temperature from 60 to 95 °C in 20 min (±0.6°/20 sec). All runs were performed on an ABI 7300 real-time PCR system (Life Technologies). For each assay, a “No Template Control” (NTC) was included.

### DNA walking

To isolate unknown sequences flanking the known sequences, a PCR-based DNA walking was performed as previously described^17–19,21,23,24^. Briefly, a target-specific primer (a) and one kind of the degenerated random tagging primer (DRT) mixes (A-D) were first used to amplify the sequences of interest. In a second and third semi-nested PCR, target-specific primers (b and c) were sequentially combined with universal tagging primers, UAP-N1 and UAP-N2 respectively, in order to increase the yield of the sequences of interest and to decrease the background. For each petunia sample (n°1–18 and n°21–23) presenting earlier a positive signal in real-time PCR for the p35S marker, 100 ng of DNA were tested using the bidirectional DNA walking method anchored on p35S. The final PCR products were visualized by electrophoresis using the Tapestation 4200 device with the associated High Sensitivity D5000 Screen Tape and reagents (Agilent) (Fig. 2, Supplementary File 1). The resulting PCR products were sequenced using the MinION platform from Oxford Nanopore technologies.

### MinION sequencing and data analysis of PCR products generated by DNA walking

For each GM petunia sample (n°1–18 and n°21–23), all PCR products generated by the bidirectional DNA walking method anchored on p35S were pooled to be purified using the AGENCOURT® AMPURE® XP Kit (Beckman Coulter Life Sciences) according to the manufacturer’s instructions. The concentration of each purified pool was measured using the Qubit dsDNA HS Assay Kits by the Qubit 3.0 Fluorometer (Life Technologies) according to the manufacturer’s instructions. For each purified pool, a sequencing library was prepared from 1 µg of DNA using the 1D amplicon by ligation sequencing kit (SQK-LSK108; Oxford Nanopore Technologies, Oxford, UK) and the 1D Native barcoding kit (EXP-NBD103; Oxford Nanopore Technologies, Oxford, UK) according to the manufacturer’s instructions. More precisely, each purified pool was initially end-repaired and dA-tailed to be subsequently ligated individually to a unique barcode. All these barcoded samples were then pooled to be ligated to adapters in order to constitute the sequencing library. As only 12 different barcodes were available, two sequencing libraries were prepared, one for the samples n°1–11 and one for the samples n°12–18 and n°21–23 (Supplementary File 8). Finally, each sequencing library was loaded into an individual MinION flow cell of the MinION sequencing device (Oxford Nanopore Technologies, Oxford, UK) to be sequenced for 48 hours. The basecalling of the generated raw data (fast5 format) was performed using Albacore (version 2.0.1). The demultiplexing of the different petunia samples, individually barcoded with a unique barcode during the library preparation, was automatically carried out during this step. The individual fastq files were extracted from the base-called fastq5 files with poretools (version 0.6.0). The raw data are available on NCBI (SRA file accession number: PRJNA523486).

The generated sequences were analyzed via a rapid workflow using CLC Genomics Workbench (Qiagen). The sequences were mapped to available petunia genomes [https://solgenomics.net/organism/Petunia_inflata/genome; https://solgenomics.net/organism/Petunia_axillaris/] at default parameters to find the transgene flanking regions. The sequences presenting a partial similarity to petunia genomes were blasted to the NCBI database to characterize the part corresponding to the transgenic cassettes. In addition, the sequence presenting no correspondence to the petunia genomes were blasted to the NCBI database, allowing to characterize the transgenic cassettes. Additional sequence analyses (alignment and creation of consensus sequences) were performed using CLC Genomics Workbench (Qiagen) (Fig. 2, Supplementary Files 2–7).

### Verification of transgene flanking regions by PCR amplification and Sanger sequencing

The identified transgene flanking region from the GM petunia samples n°1–3, n°5, n°7–9, n°11–14, n°16–18 and n°21–23 observed with the MinION sequencing platform were verified by PCR. For this PCR confirmation assay, a forward primer (Junction-F) and a reverse primer (Junction-R) were designed using the software Primer3 to anneal respectively to the sequences from the petunia host and the transgenic cassette in order to amplify by PCR a DNA fragment of 402 bp (Fig. 2, Table 1, Supplementary File 6). In addition to these petunia samples, this PCR confirmation assay was applied on the petunia samples n°4, n°6, n°10, n°15 and n°19–20. An NTC also was included.

For all PCR assays, a standard 25 µl reaction volume was applied containing 1X Green DreamTaq PCR Master Mix (ThermoFisher Scientific), 250 nM of each primer (Eurogentec) and 5 µl of DNA (5 ng/µl). The PCR program consisted of a single cycle of 1 min at 95 °C (initial denaturation) followed by 35 amplification cycles of 30 sec at 95 °C (denaturation), 30 sec at 50 °C (annealing) and 1 min at 72 °C (extension) and finishing by a single cycle of 5 min at 72 °C (final extension). The run was performed on a Swift MaxPro Thermal Cycler (Esco). The PCR products were visualized by electrophoresis using the Tapestation 4200 device with the associated High Sensitivity D1000 Screen Tape and reagents (Agilent) (Supplementary File 5). The sequencing of the PCR products, earlier purified using USB ExoSAP-IT PCR Product Cleanup (Affymetrix) according to the manufacturer instructions, was performed on a Genetic Sequencer 3130XL using the Big Dye Terminator Kit v3.1 (Applied Biosystems). The resulting sequences were analyzed using CLC Genomics Workbench (Qiagen) (Fig. 2, Supplementary Files 5,6).

The sequences of the transgene flanking regions observed with the MinION (Fig. 2, Supplementary File 3) and Sanger platforms (Fig. 2, Supplementary File 5) were compared by alignment using CLC Genomics Workbench (Qiagen) and Nucleotide BLAST NCBI (Supplementary File 6).

## Supplementary information

supplementary files

## Data Availability

All data generated or analyzed during this study are included in this published article [and its Supplementary Information Files] or is available from the corresponding author.
