# Supplementary material for: MinION sequencing technology to characterize unauthorized GM petunia plants circulating on the European Union market
Source: Sci Rep. 2019 May 9;9:7141. doi: 10.1038/s41598-019-43463-5 (PMC6509135; doi:10.1038/s41598-019-43463-5)
Supplement: Supplementary file 1 — supplementary files [file 41598_2019_43463_MOESM1_ESM.docx]

**MinION sequencing technology to characterize unauthorized GM petunia plants circulating on the European Union market**

**Marie-Alice Fraiture ^a^** (Marie-Alice.Fraiture@sciensano.be)

**Gabriella Ujhelyi ^b^** (UjhelyiG@nebih.gov.hu)

**Jaroslava Ovesná ^c^** (ovesna@vurv.cz)

**Dirk Van Geel ^a^** (Dirk.VanGeel@sciensano.be)

**Sigrid De Keersmaecker ^a^** (Sigrid.DeKeersmaecker@sciensano.be)

**Assia Saltykova ^a^** (Assia.Saltykova@sciensano.be)

**Nina Papazova ^a^** (Nina.Papazova@sciensano.be)

**Nancy HC Roosens ^a,*^** (nancy.roosens@sciensano.be)

**^a^** Sciensano, Transversal activities in Applied Genomics (TAG), J. Wytsmanstraat 14, 1050 Brussels, Belgium

**^b^** National Food Chain Safety Office, Mester utca 81. H-1024 Budapest, Hungary

**^c^** Crop Research Institute, Department of Molecular Genetics, Drnovská 507, 161 06 Prague, Czech Republic

* Corresponding author: Nancy Roosens (nancy.roosens@sciensano.be); Sciensano, Transversal activities in Applied Genomics (TAG), J. Wytsmanstraat 14, 1050 Brussels, Belgium. Tel: 00 32 (0) 2 642 52 58. Fax: 00 32 (0) 642 52 93

**Supplementary materials**

**Supplementary file 1: Visualization of PCR products from the bidirectional DNA walking method anchored on p35S (p35S-F and p35S-R directions) applied on GM petunia samples (n°1-18 and n°21-23).** The molecular-weight size marker is going from 15 to 10 000 bp.

| **p35S-F DNA walking method** |
| --- |
| With DRT A primer |
| 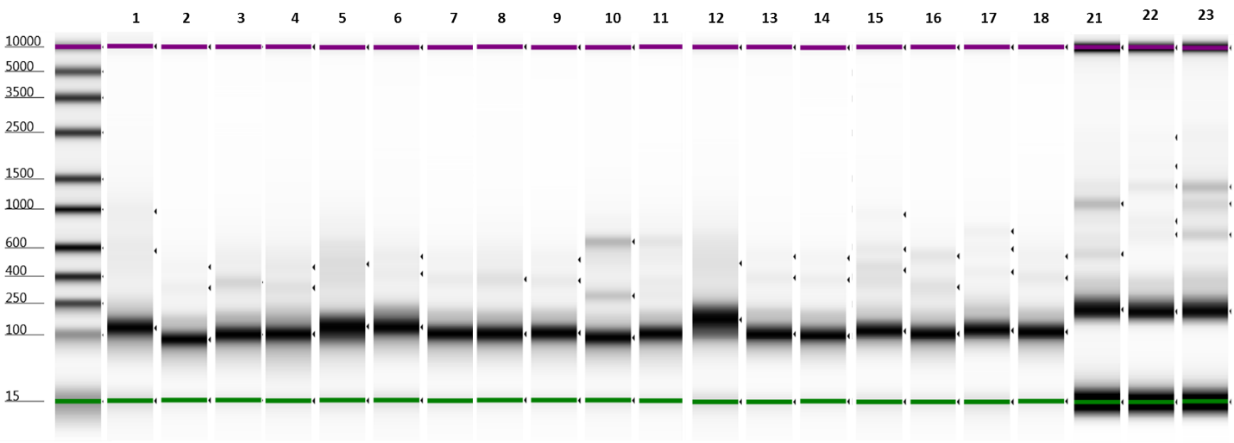 |
| With DRT B primer |
| 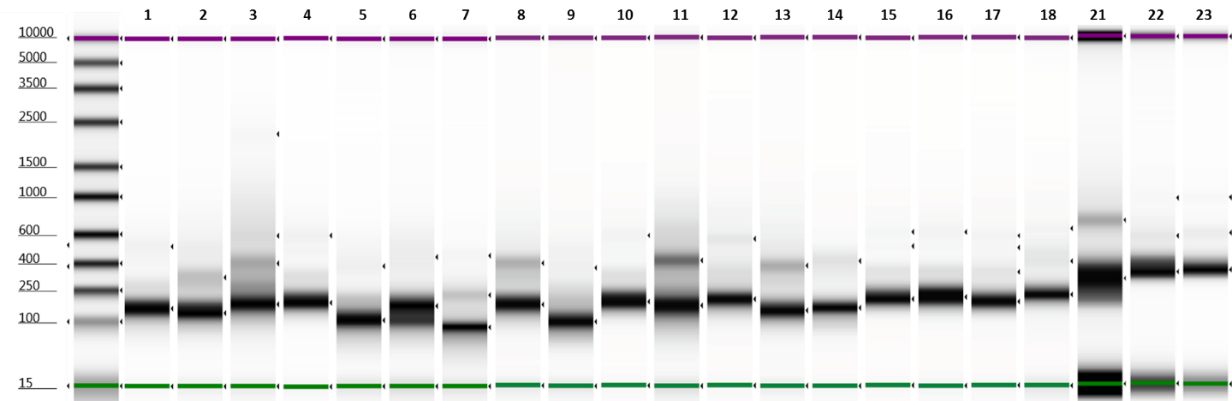 |
| With DRT C primer |
| 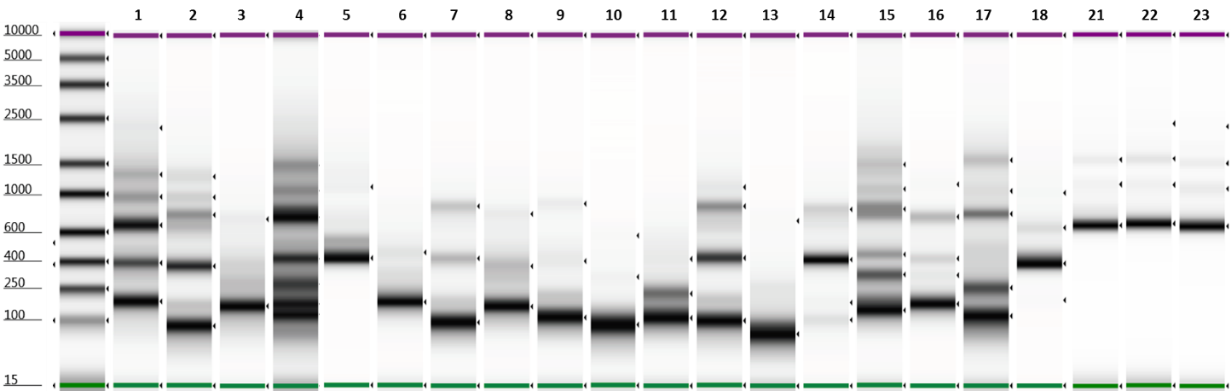 |
| With DRT D primer |
| 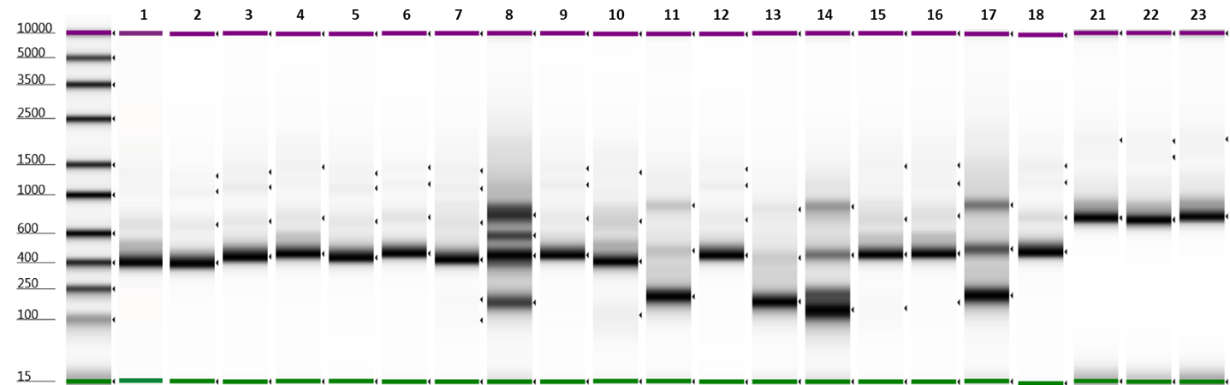 |
| **p35S-R DNA walking method** |
| With DRT A primer |
| 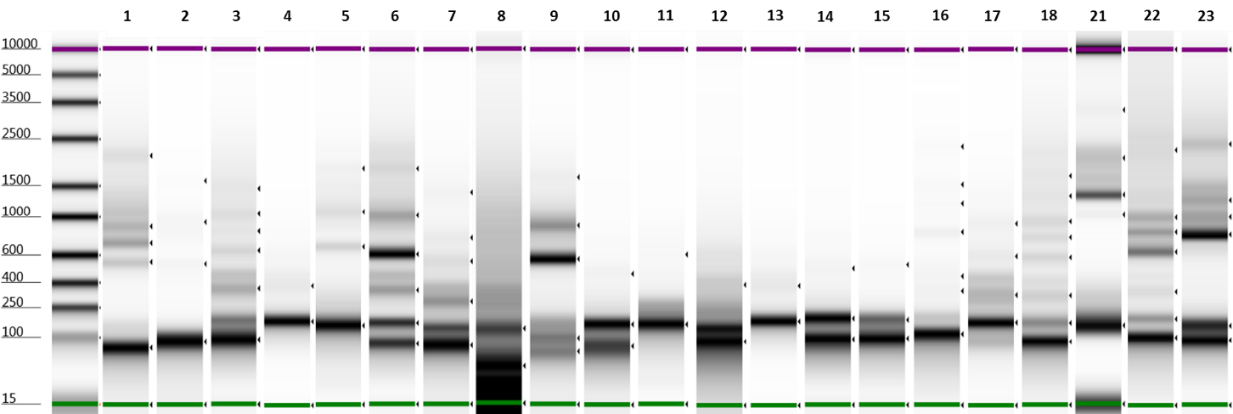 |
| With DRT B primer |
| 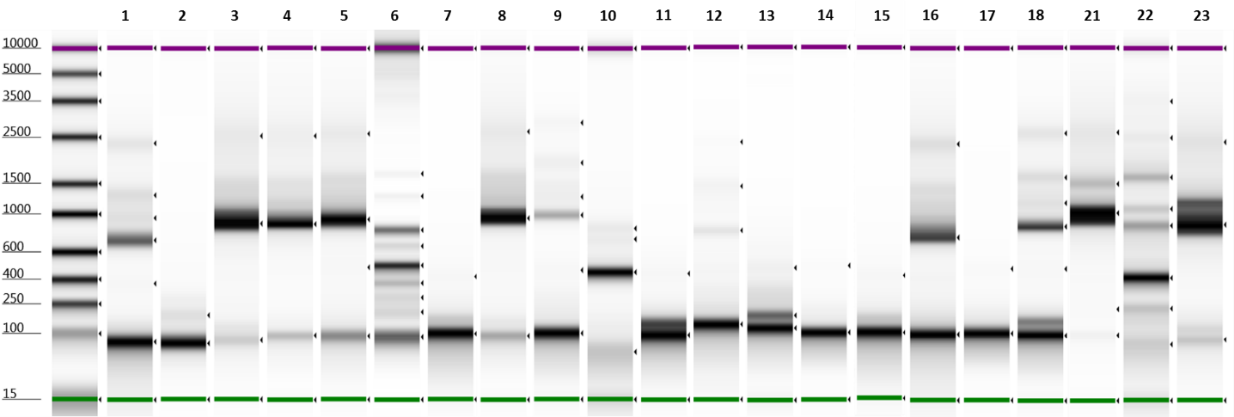 |
| With DRT C primer |
| 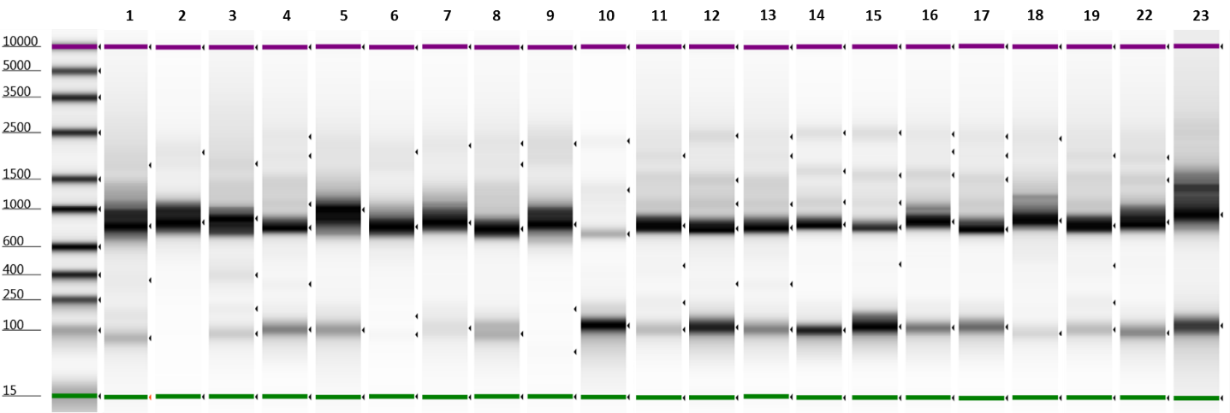 |
| With DRT D primer |
| 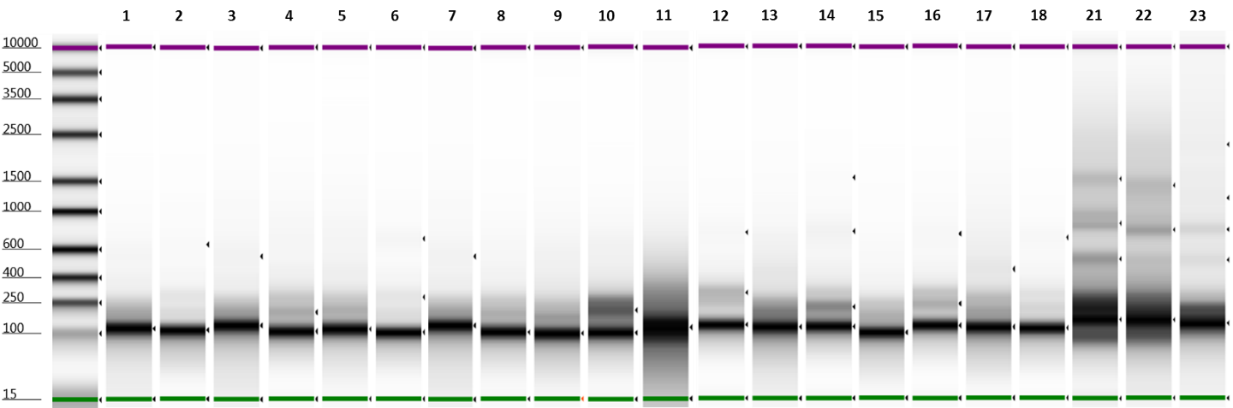 |

**Supplementary file 2: A part of the transgenic cassette, including the p35S, uncharacterized vector part and the maize *A1* gene, identified from the MinION sequencing data.** (A) This sequence is a consensus based on all corresponding sequences observed in all samples suspected to contain GM petunia (n°1-18 and n°21-23). The sequence corresponding to p35S is indicated in bold while the sequence corresponding to the maize *A1* gene is dotted underlined. (B) Alignment of the part of the maize *A1* gene observe in the point A to a part of the maize *A1* gene reported by Schwarz-Sommer et al., 1987 [25] (NCBI accession numbers: X05068.1 and NM_001158995.2). 97% of similarity are observed.

| **A** |
| --- |
| **CACTATCCTTCGCAAGACCCTTCCTCTATATAAGGAAGTTCATTTCATTTGGAGAGGACAGGGTACCCGGGGATCC**TCTAGAGAATTCCAGCTGCTCACTCAGTCCTGCGCAAGAGCTCGCTCTCGGAGAAAAAACGCGGGAGGCGATAATGGAGGAGGTGCCGGTGCGAGCGAGAAAGGGACGGTGCTGGTCACGGGGCGTCGGGCTTCGCCGGCTCCTGGCTCGTCATGAAGCTCCTCCAGGCCGGCTACACCGTCCGGGCGACCGTGCGCGATCCCGCGAA |
| **B** |
| **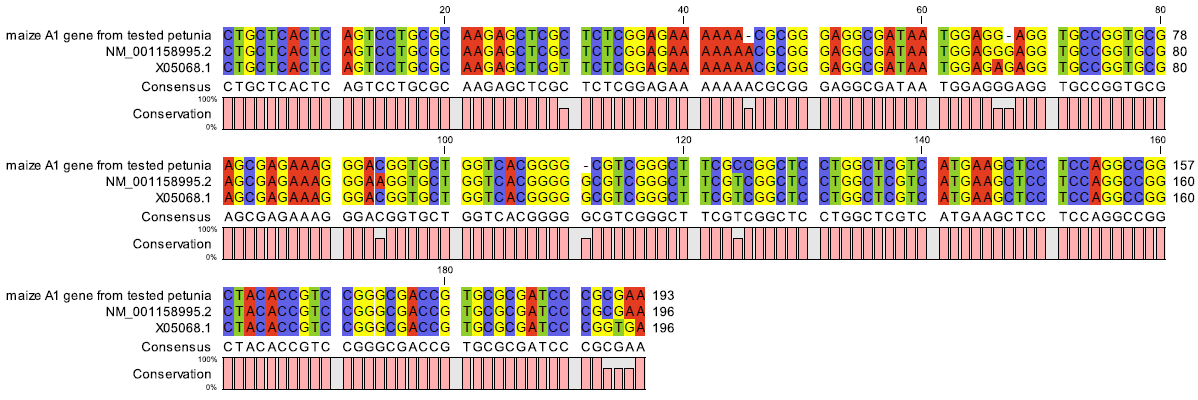** |

**Supplementary file 3: Transgene flanking regions identified from the MinION sequencing data.**

The petunia part is indicated in lower case while the transgenic cassette, containing *bla* (underlined), uncharacterized vector part and p35S (in bold), is indicated in upper case.

For the samples associated to an asterisk (n°4, 6, 10, 15), only the transgenic cassette was identified from the MinION sequencing data while the same transgene flanking region, similar than the ones from the samples n°1-3, n°5, n°7-9, n°11-14, n°16-18 and n°21-23, was observed by the PCR confirmation assay (Table 2, Supplementary file 5).

| **Samples** | **Sequences of identified transgene flanking regions** | **Size** |
| --- | --- | --- |
| **1** | attacattaatacgccatttctaccggtggtggtggtggtgaaacaccacgtcacgtttacctCAGAAGTAAGTTGGCCAGTGTTATCACTCATGGTTATGGCAGCACTGCATAATTCTCTTACTGTCATGCCATCCGTAAGATGCTTTTCTGTGACTGGTGAGTACTCAACCAAGTCATTCTGAGAATAGTGTATGCGGCGACCAGTTGCTCTTGCCCGGCGTCATCACGGGGATAATACCGCGCCACATAGCAGAACTTTTTAAAAGTGCTCACGCTATTGAAAACGTTCTTCAAAGGAAACTCTCAAGGATCCACCGCTGTTGAGATCCAGTTCGATGTAACCCACCTCAGCAATCCAACTGATCTTCAGCATCTTTTACTTCACCAGCTGCTCTGGGTGAGCAAAACAGGAAGGCAAAATGCCGCAAAAAGGGAATATGAATATTGACGGAAATGTTGAATACCTTTCATACTTCCTTTCACATATTGAAGCATTTATCAGGGCTACTGTCTCAGAGCGGATACACATTTGAATGTATTTAGAAAAATAAACAAATAGGGTTCCGCGCACACCTCGAAAAGCGCCACCCGACGTCCAAGAAAGTCCATTGCCATCACAGCGACAACCTACAAAAATAGCGTAACACGAGGCCCTCGTCTCAAGAATC**CTACGGAGTCAAAGATCCAAACAGAGGACCCAACAGTAGAATCCTTGCCGTAAAGACTGGCGAACAGTTCCATATACAGAGTCTTCTACGTGACTCCAATGACAAGAAGAAAATCTTCGTCAACATGGTGGAGCACGACACGTTTGTCTACCCTTGTAATACCAAAGAGGGTTAGTCTCAGAAGACCAAAGGGCAATTGAGACTTTTCAACAAAGGGTGGTTGTCCGAAACCTCCTCGATTCCATTGCCCAGCTATCTGTCACTTTTATTGTGAAGATAGTGGAAAAGGAAGGTGGCTCCTACAAATGCCATCATTGGGTGAGATAAAGGAAAGGCCATCGTTGTAAGATGCTCTGCCGACAGTGGTCCCAAAGATGGACCCCCACCCACGAGGAGTATCAGGGAAGAAGACGTTCCAACCACGTCTTCAAAGCAAGTGATTGATGTGA** | 1120 bp |
| **2** | cgggcgtctgtggtacaaccgccttagcgaaattaccctactgaaagaagaatttatagaaatgatcagtttgtcgttgtgtctttataaagggtctggatctgagtttgttataattgctgtgtatgttgatgacttgaattattggaactccacagagattccaaaggcagtgaactatttgaagaaaatttgaaatgaaagatcttggaaagacaaattttgtcttggtcttcaaattgaggcatttgataaatggaattttacccatcaatcaacatacactggaaaagattttaaagagattttatatggataaatctcatccctttgagtactccgatggtcgtgagatcgcttgacataagtaaagatcatttcgccctcatgaaaatgatgaaaagattcttggtgctgaaacaccatatgccagtgcaattaggcattaatgtatcttgctaataatacccgatgaacattgctttttctgtaaacttattagcaaagatttagttcttccccgacacaagacactggaatggtatcaaacatttattcagatacctccgaggaacaatcgacaaaggtttgttttattcaaagtgaatccaacttgccactaattggttatgCAGAAGTAAGTTGGCCGCAGTGTTATCACTCATGGTTATGGCAGCACTGCATAATTCTCTACTGTCATGCCATCCGTAGATGCTTTTTTCTGTGACTGGTGAGTACCCAACCAAGTCATTCTGAAATAGTGTATGCGGCGACCGGAGTTGCTCTTGCCCGGCGTCAACACGGAATAATACCGCGCCACATAGCAGAACTTTAAAAGTGCTCATCGGTGAAAACGTTCTTCGGGTGAAAACTCTCAAGGATCTTACCGCTGTTGAGATCAGTTCGATGTAAACACTCGATGCACCCAACTGATCTTCAGCATCTTTTACTTTTCACCAGCGTTTCTGGGTGAGCAAAAACAGGAAGGCAAATGCCGCAAAAAGGGAATAAGGGCGACACGGAAATGTTGAGAATACTCGCACTCTCTTCCTTTTCAATATTGTTGAAGCATTTATCAGGGTTATTGTCTCGCAGGCGGATACATATTTGAATGTATTTAGAAAATATAAAATAGGGGTTCCGCGCACATTTCCCGAAAAGTGCCACCTGACGTCTAAGAAACCATTATTATCATGACATTAACCTATAAAATAGGCGTATCCCACGAGGCCCTTTCGTCTTCAAGAATTC**CCATGGAGTCAAAGATTCAAATAAGGACCTAACAGAACTCGCCGTAAAGACTGGCGAACAGTTCATACAGAGTCTCTTACGACTCCAATGACAAGAAGAAAATCTTCGTCCAACATGTTGGAGCTGACACCTTGTCTACTCGAGAAATATCAAAGATACGATCTCCAGAAGACCAAAGAGACAGGTGAGACTTTTCAACAAGGTAATATCCGGAAACCTCCTCGGATTCCATTGCCCAACTATCTGTCACTTTATTGTGAAGATAATTGGAAAAGGAAGGTGGCTCTACAAATGCCATCATTGCGATAAGGAAAGGCCATCGCTTGAAGATGCCTCTGCCGACAGTGGTCCCAAGATGGACCCCACCCACGAGGAGCGTCGTGGAAAAGACGTTCAACCACGTCTTCAAAGGCAAGTGGATTGATGTGATAAGAT** | 1686 bp |
| **3** | ttgctaataatacccgaccagacattgctttttctgtaaacttattagatttgatttccgacacaagacactggaatggtatcaaacatttattcagatacctccgaggaacaatcgacaaaggtttgttttattcaagtgaatccaacttgccactaattggttatgCAGAAGTAAGTTGGCCGTGTTATCACTCATGGTTATGGCAGGCACTGCATAATTCTCTTACTGTCATGCCATCCGTAAGATGCTTTTCTGTGACTGGTGAGTACTCAACCAAGTCATTCTGAGAATAGTGTATGCGGCGACCGAGTTGCTCTTGCCCGGCGTCAACGGGATAATACCGCGCCACATAGCAGAACTTTAAAAGTGCTCATCATTGGAAAACGTTCTTCGGGGCGAAAACTCTCAAGGATCTTACCGCTGTTGAGATCCAGTTCGATGTAACCCACTCGTGCACCAACTGATCTTCAGCATCTTTTACTTTCACCAGCGTTTCTGGGTGAGCAAAACAGGAAGGCAAAATGCCGCAAAGGGAATAAGGGCGACACGGAAATGTTGAATACTCATACTCTTCCTTTTCAATATTATTGAAGCATTTATCAGGGTTATTGTCTCATGAGCGGATACATATTTGAATGTATTTAGAAAAATAAACAAATAGGGGTTCCGCGCATTTCAAAAGGGGACCACCCTGACGTCTAAGAAACCATTATTATCATGACATTAACCTATAAAAATAGGCGTATCACGAGGCCCTTTCGTCTTCAAAAATTC**CCATGGAGTCAAAGATTCAAATAGAGGACCTAACAGAACTCGCCGTAAAGACTGGCGAACAGTTCATACAGAGTCTCTTACGACTCAATGACAAGAAGAAAATCTAACATGGTGGAGCACGGAATCAAAGATACAGTCTCAAGAAGACCAAAGGGCAATTGAGACTTTTCAACAAAGGGTAATATCCGGAAACCTCCTCGGATTCCATTGCCCAGCTATCTGTCACTTT** | 1006 bp |
| **4*** | ATGCGGCGACCAGTTGCTCTTGCCCGGCGTCATTACGGGATAATACCGTTACATAGCAGAACTTTAAAAGTGCTCATTTATTGAAAAACGTTCTTCGGGGCGAAAACTTCAAGGATCTTACCGCTGTTGAGATCCAGTTCGATGTAACCCACTCGTGCAATCTAACTGATCTTCAGCATCTTTTACTTCAACAGCGTGTTTCCAAGAGCAAAACAGGAAGGCAAAAATGCCGCAAAAAAGGGAATAAGGGCGAATAATGGGAAATGTTGAATACCATACCTTCCTTCAATATTATTGAAGCATTTATCAGGGTTATTGTCTCCCATGAGCGGATACATATTTGAATGTATTTAGAAAAATAAACAAATAGGGGTTCCGCGCACATTTCCGAAAAGCTTGCCCGACGTCTAAGAAACCATTATTATCAGCGACATTAACCCCTATAAAAATAGGCGTATCACGAGGCCCTTTCGTCTCTCAAGAATTC**CTACGGAGTCAAAGATTCAAATAGAGGATCCACAGAACTCGCCGTAAAGACTGGCGAACAGTTCATACAGAGTCTCTATGACTAGACAAGAAGAAAATTCGTCAACATGGTGGGATAATGACACGCTTGTCTACTTCCAAAATATCAAAGATACAGTCTCAGAAGACCAGCAAGTACTGAGATTCGCCAACAAAGGGTAATATCCGGAAACCTTCTCGATTCCATTGCCCAGCTATCTGTCGCTCTTTATTGTGAAGATAGTGGGAAAAGGAAGGTGGCTCCTTGGCCAAAAGCCATCATTGCGATGCCAGCGAGCGGCCATCGTTGAAGATGCCTTGCCGACAGAGGTCCTGCAAAGATGGACCCCCACCCACGAGGAGCATCGGAAAAGAAGACGTTCCAAACCACGTCTTCAAAGCAAGTGGATTGAT** | 918 bp |
| **5** | tggaaatcggtatcaaacatttattcagatacccgagaaacaatcgacaaaaaaggtttgttttatcaagtgaatccatcctgccactaattggcgaagttatgCAGAAGTAAGTTACCGCAGTCACCGCTGGTTATACGCACTTCGTGGTATACCTCTACTGTCGCTGCCATCCGTAAGATGCGCTTTCTGTGACTGGTGGGTACCTTTCAACCAAGTCATTCTGAGAATAGTGTGTATGCGGCGACCAGTTCTTCTGCCCGGCGCCAACAATGGAGTTGGGTCGCGCCACAGCAGCAGAACTTTATAAGTGCTCATCATTGGAAAACGTTCTTTCGGGGCGAAAAATTAATAGATCTACTGCTGTTGAGAGATCCAGTTCGATGTAACCTGTCGCACTCAAAAATCTAATCATTGATCTTCGTAGCATCTTTTACTTTCTACCAAGCGTTTCTGGGTAGAGTTAAAAACAGGAAGCCGCCGCAAAAAGGAATAAGGGCGACACGAAACGTTGAACATACCATACCTTCCTTTTCCAACAAGCCATTGAAGAATGGCATCAGGCTATTGTCCCATGAGCGGATACATACTGAACGTATCAGAAAACAAACAAACAGTTCCGCGCTAATACTTCCCCGAAAAGTGCCAAGCACCTGGACGTCTGGGTTAAAACCATTATTATCATGACGTTAAACCCATAAAATAGGCGCATCACGAGGCCCTTCTTTGCCCTCAAGAATTC**CCCATGAGTCAAAGATTCAAATAGAGGACTCAACAGAACCACGTAAAAAGACTGGCGAACAGTTCATACAGAGTCTCATGACTCCAACACGTTAGCCAGAAAATCTTCAGTCAACATGGTGGAGCACGACACGCTGGTTCACCCAAAATATCAAAAGATACAGTCCAGAAGACCAAAAGGGCAATTGAGGACCAACAAAGGTACATCCGAAACCTCGGACGAACGATGTCTGTGCCATTCGTCACTTTATTGTGAAGATAGTGGAAAAGGAAGGTGGGCTCCTACAAATGCCATCATTGCGATAAAGCAGGAAAGCCACGTTGAAGATGCCTCTTGCCGACAGTGGTCATCCCAAGCAAAAGATGGATCCCACCCACGAGGAGACATCGTGGAAAAAAGAAGACGTTCCAACCACGGCCCTCAAAGCAAG** | 1174 bp |
| **6*** | CAGTTCGATGCCACTCGTTACCCAACTGATCTTCAGCATCTTTACTTTCACCAGCGTTTCTGGGTGAGCAAAACAGGAAGGCAAAATGCACAAAAGGAATAAGGACGACACGAATGTTGAATACTCATACTCTTCCTTTTCAATATTGAAGCATTTATCAGGGTTATTGTCTCGAGCGGATACATGTTTGAATGTGTTAAGCAAAATAGGGTTGCTTTTGAAAAGATGCCACCTGACATCTAAGAAACCATTATTATCATGGCATTAACCTATAAAATAGGCGTATCACGAGGCCCTTTCGTCTTCAAAGAATTCC**CATGTGGAGTCAGATTCAAATGAAGGGACCTAACAAGGAACTCGCCATGAGGCCTTTAGCGAACAGTTCATACAGAGTCTCTTACGACTCGACAAAGAAGAAAATCTTCGTCAACATGGTGGAGCACGACACGCTTGTCTACTCAAAAATATCAAAGATACGAAGTCTCAGAGACCAAAGGGGCAATGAGACTTTTCAACAAAAGGGTAATATCGAGCCTCCTCGGATTCCATTGCTTGCAGCTTATCTGTCACTTTATTGTGAAGATGAATTGGAAAAGGAAGGTGGCTCCCTACAAATGCCATCATTGCGATAAAAGGAAGAGACCCATCGTTGAACGCCTCTGCCGACGAGGAAGTGGTCCCCAAAGACCCACCCACATGAGAACATCGTGTACAGAGAAGA** | 721 bp |
| **7** | cgtggaacaatcgacaaaaggtttgttttattcagagtgaatcaacttgccactaattggttatgCAGAAGTAAGTTGTAGCCGCAGTGTTATCACTCATGGTTATGGCAGCACTGCATAATTCTCTCTTACTGTCATGCCATCCGTAAAGGAGTCTTTCTGTGACTGAAATTCAGGTACTCAGCAAGTCATTCTGAGAATAGTGTATGCGGCGACCGGATTTGCTCTTGCCCGGCATCAACACGGGATAATACCGCGCCACATAGCAGAACTTTAAAAGTGCTCATCATTGGAAAACGTTCTTCGGGGCGAAAACTCTCAAGGATCTTACCGCTGTTGAGATCCAGTTCGATGTAACCCACTCGTGCGCAACTGATCTTCAGCATCTTTACTCTCACCACCAGCGTTTTCTGGGTGAGCAAAAACAGGAAGGCAAAATGCCGCAAAAAGGGAATAAAAGGGCGACACGGAAATGTTGAATACTCGCCTCTTCCTTTCAATATTATTGAAGCATTTATCAGGGTTATTGTCTCATGAAGCGGATACATATTTGAATATTTAGAAAAATAAACAAATAGGGTTCCGCGCACATTTTCCCGAAAAGTGCCACCTGACGTCTGAAACCATTATTATCATGACATTAACCTATAAAATAGGCATCACGAGGCCCTTTCGTCTTCAAAATTC**CCATGGAGTCAAAGATTCAAATAGAGGACCTAACAGAACTCGCCGTAAAGACTGGCGAACAGTTCATACAGAGTCTCTTACGACTCAATGACAAGAAGAAAATCTAACATGGTGGAGCACGGAATCAAAGATACAGTCTCAAGAAGACCAAAGGGCAATTGAGACTTTTCAACAAAGGGTAATATCCGGAAACCTCCTCGGATTCCATTGC** | 898 bp |
| **8** | tcacgctacctccgaggaaccttcgacaggttttgttttattcaaagtgaatccagcttacccactaattggttatgCAGAAATGAGATTGGCCGCAGTGTTATCCTCATGAATTTATGGCAGCACTGCATAATTCTCTTACTGACATACCATCCGTAAGATGCTTTTTCTGTGACTGAGTGAGTACTCAACAAAGTCATTCTGAGAATAGTGTATGCGGCGACCGAGTTGCTCTTGCGGCGTCAGCACGGGATAATGCCGCGCCGCATGGCAGAACTTTTAAAGAAGTGCTTTCATCGTTGCGGAAGCGTTCTTCGGGGCGAAAACTCTCAAGGATCTTGCAACGATGAGGATCGGTTCGATGTAACTTGCTCGTGCTTGGCGGTCTTCCCGTATCTTTACTTTCACCAGCGTTTCTGGAAGTGAGAGCAAAAACAGGAAAGCAAAATACAAAAGTCAAGAGCGGCAAATGTTGAATACTCTTTTCTTCCTTTTCAATATTATTGAAGCTATCAGAGATTATTATCCTCGCAGAAGCGGATACATATTTGAATGTATTAGAAATAAACAAATAAGTTCCATTCCCAAAAAGTACCTGAAGCGTCTAAAGAAACCATTATTATCATGACATTAACCTATATAAAAATAGGCGTATCACGAGGCCTTTCGTCCTCAAGAATTC**CCATGAGTCAAAAGATTCAAATAGAAGGGATTCCCTAACAGAACTCGCCGTAAGGAGCCCGGCGAGCAGTTCATACAGAGTCTCTTACGACTCAATGACAAGAAGAAAATCTTCGTCAACACATGGTGGAGCACGACACGCTTGTCTACTCAAAATATCAAAAGATACAGTCTCAGAAGACCAAAGGGCAATTGAGACTTTTTCAACAAAGGTAATATCCGGAAACCTCCCTCAGTTCCATTGCCCAGCTATCTGTCACTTGCTATTGTGAAGATAGTGGAAAAGGAAGGTGCTCCCCTACAAAAACGCCATCGTTGCGATATAAAGGAAGACCATCGTTGAAGATGCCTCTGCCGACAGTGGTCAAAGATGGACCCCCACCCACGAAGACATCGTGGAAAAGAAGACGTTCAACCGTCTTTACAGAAGCCAGTGGATTGATGTGATATTAAGGTG** | 1138 bp |
| **9** | aattagagatatcgtatcttgctaatactggctcgaccagacattactgcttgcaaacttattagcaagattgaagttcttccccgacacaaagacaattgaatggtatcaaacatttattcagatacctccgaggaacaatcgacaaaggtttgttttatctgcgaatccaacttgccactaattggttatgCAGAAGTAAGTTGGCTGCAGTGTTATCAATACTATGGTTATGGCAGCACTGCATAATTTCTACCGTACCGTCATGCCATCCGTAAGATGCTTTTCGTGACTGCGAGCACCAACCAAGCCATTCTGAGAATAGTGTATGCGGCGACCGAGTTGTTTCGCCTCGGCGTCAACACGGACAATACCGCGCCACAGCAGAACTCTTTAAAGGTGCTCATCATTGGAAAACGTTCTTCGGGCGAAAACTCATGATTCACCGCTGTTGAGATCCAGTTCGACGTAACCCACCGTAATCTAACTGGATCTTCTTCAGCATCTTTACTTCACCAGCGTTTCTGGGTGAGCAAAACAGGAAGGCAAAATGCCGCAAAAAGGGAATAAGGGCGACACGGAAATGCTGAATACTCATACCTTTCCTTTTCAATATTATTGAAGCATTTATCAGGGTTATTGTCTCATGAGCGGATACATGGCTTGAACGTATTTAGAAAAATAAAACAAATAGGTTCCGCGCATTTCCTCGAAAAGTGCCACCCGACGTCTAAGAAAACCATTATTATCATGACATTAACCTATAAAAATAGGCGTATCACGAGGCCTCCTTTCGTCTTCAAGAATTC**CCACGGAGCCCAAAGATCCAAATAGAGGACCAACAGAACCCGCCGCAAAAGACCGGCGAACAGTCTCATTAAGAGTCCTTACGACCACCATCAGCCAGCACCTTCGTCAACACGGCGGCTAACGACACGCCTGTCCAATTCCAAAAATAACACAAACTCGCAAAATAACTCAAAGGGCAATTGAGACTTTTCAACAAAGGGTAATATATCCGGAAACCTCCTCGGATTCCAGAGCTGCTCCAGCTTATCTGTCACTTTATTGTGAAGATAGTGGTGCTGAAGGTGGCTCCTGCCAAATGCCATCATTGCGATAAAGGAAAGGGCCATCGTTGAAGATGCCTTCTGCCGGACAGTGGTCCGGGAAGATGGACCCCACCCACGGGAGCATCAGGGAAAAGACGTTCCAACCACGTCTTCAAAGCAAGTGATTGATGTGATA** | 1248 bp |
| **10*** | TAGCGAATCGGCGTCCGCTTACCTCCGGGGCGTCATCACGGGATAATACCGCGCCACATAGCAGAATTGCTTAAAAGTGCTCATCATTGGAAAACGTTCTTCGGGGCGAAAACTCTCAAAGGATCTTACCGCTGGTGAGATCAGTTCGATGTAACCACTCGTGCATAGCGATCTTCCAGCATCTTTTACTTTCACCAGCACGTTTCTGGGTGAAGGCAAAACAGGAGAAATGCCGCAAGGGAATAAGGGCGACACGGAAATGTTGAATGCTCATACTTTAACTTCCTTTTTTCACGTATTATTGAAGCATTTATCAGAGTTATTGTCTCATGAGCGGATACATATTTGAATGTATTTAGAAAAATAAACAAATAGGGGTTCCGCGCATTTCCCGAAAGTGCCACCTGACGTCTAAGAAACCATTATTATCATTAACTATAAAAATAAGGCGTATCCACGAGGCCCTTTCGTCTTCAAATTC**CCATGGAGTCAAAGATTCAAATAGAGGACCTAACAGGACTCGCCGTAAAGACTGGCGAACAGTTCATACCGAGTCTCTTACGACTCAATAACAAGAAGAAAATCTTTAACGTCAACATGGTGGAGCACGACACGCTTGTCTACTCCAAAAATATCAAAGATACAGTCTCCAAGAAGACCAAGGGCAATTGGGACTTTCAACAAGGTAATATCCGGAAACCTCCTCGAAGTTCCATTGCCAGCTATCTGTCACTTTATTGTGAAGATAGTGGAAAGGAAGGTGAGCTCCTACAAATGCCATCATTGCGATGAAGGCCATCGTTGAAGATGCCTCTGCCGACAGTGGTCCCAAAGATGGACCCCACCCACGAGGAGCATCATTGAAAGAAGACGTTCAACCGACGTCTTCAAAAGCAAGTGGATTGATGTGATA** | 913 bp |
| **11** | caattgctgcagtgaagcaagccgggtcgcgaaaggttaataattggcGGTTATGGCAGCACTGCATAATTCTTTACTGTCATGCCATCCGTAAGATGCTTTCTGTGACTGGTGAGTACTAAATCCGTTGAGAATAGTGTATGCGGCGACCGAATTGCTCTTGCCCGGCGTCAACACGATAATACCGTGCCACATATATGAACTTTAAAAGTGTTCATCATTGGAAAACGTTCGGGCGAAAACTTCCGCCAAGATCTTTACCGCTGTTGGAGATCCAGTTCGATGTAACCGCACTTCGTGCACCCAACTGGATCTTTCAGCATCTTTTACTTTCACCAGCGTTTCTGGGTGAGCTAAAGAAAGGAAGGCGAAATGCCGCAAAAAGGAATAAGGTAGCGACACGGAAAATGTTGAATACTCATACTCTTCCTTTTTCAATATTATTGAAGATGATCAGGGTTATTGTCTCGCCGAGCGGATACATTTGAATGTGATGAAAATAAACAAGCTCGCGCACATTTCCCCGGAAAGTTTTCACCACCTGACGTCTAAGAAACCATTATTATCATGACATTAACCTGGCGTATGCATCACGAGGCCCTTTCGTCTTCAAGAATTC**CCATGGAGTCAAAGATTCAAATAGAGACCTAACAGAACTCTCGCTGTAAAGACTGGCGGCGAACAGTTCATACAGAGTCTCTTACGACTCAATGACAAGAAGAAAATCTTCGTTTCTAACATGGTGGAGCACGACACGCTTGGGTTCATCTAAAAATATCAAAGATACAGTCCAGGAAGACCAAAGGGCAATTGAGACTTTTCAACAAAGGGTAATATCCGAAACCTCGGATTCCATTGCCCAGCTATCTGTCACTTTTATTGTGAAGATAGTGGAAAAGGAAGGTGTAATTCCTACAAATGCCATCATTGCGATATGAGCGGCCATCGTTGAAGATGCCTTCTGCCGACAGTGGTCCCAAAGACGGACCCCCACCACGAGGAGCATCGTGGAGAAAGAAGACGTTCCAACCACGTCTTCAAAGCAAGTGGATTGGATGCGAT** | 1062 bp |
| **12** | ctccgtggaacaatcgacaaaaggtttgttttcattcaagtgaatccaactttgccaagccaattggttatgCAGAAGTAGTTGGCCGCAGTGCTTTACCACCACGGCTGTGGCAGCACTGCATAATTTCTTTACTGTCATGCCATCCGTAAGATGCTTTCTGTGACTGGTGTAGTACCAACCAAGTCATTCTGAGAATAxTGTGATGCGGCGACCGGAGTTGCTCTTGCCCGGCGTCACGGGACAATACCGCGCCACATAGCAGAACCTTTAAAAGTGCTCATCATTGGAAAATGTTCTTCGGGGCGAAACCGCGATCTTACCGCCGTTGAGATCCAGTTCGATGTAACCCCACCGTGCACCCAACTACATAAATTTTACTTTCACCAGCGTTTCTGGGTGAGCAAGAACAGAGGGAAGGCAAAATGCCGCAAAAAAGGGAATTAAGGGCGACACGAAATGTTGAATACTCATACTCTTCCTTTTTCAATATTATTGAAGCCATCATCAGGGTTATTGTCTCATAGCGGATACATATTTGAAATGTATTAGGAAATAAAACAATAGGGTCTCCTGCACATTTCCGAAAAGTACCACCTGGACGTCTAAGAAACCATTATTATCAATGACATTAACTTATAAAGAGGCGGTTTTGCCGAGGCCTTTGGCCTTGCAGAATTC**TGGCGACAAAGATTCAGATAGAGGACTCCAACAGAACTCGCCGTAAAGACTGGCGAACAGGTTCATACAGAGTCTCTTACGACCAATGACAAGCAAGAAAATCTTCGTCAACATGGTGGAGCACGACACGCTTCGTCTACTCCAAAATATCAAAGACAGTTCCCAGAAGACCAAAGTGGGCAATGAGACTTTCAACAAAGGGTAATATCCGGAAACCTCCTCGGATTCCATTGCCCAGCCATTCGTCACTTTATTGAAGATAGTGGTGCGGTGGCTCCTACAAATGCCATCATTAAGGGATAAAGGAAAGGCCATCGCTGAAGATGTTCTCTGCCGACAGTGAAGTCCGCAAAGACGGACTCCCACCCACGAGGAGCATTTCCATGTGGAAAAAGAAGACGTTCCAACCAATGTCTTCAAAGCAAGTGGATTTGATGTGATAA** | 1124 bp |
| **13** | acctccggggaacaatcgacaaaaggtttgttttgttagaatccaacttgcactaattggttatgCAGAAGTAAGTTGGCCGAGTGTTATCACTCATGGTTATGGCAGCACTGCATAATTCTCTTACTGTCATGCCATCCGCTAAGATGCTTTTTCTGTGACTGGTGAGTACTCAATAAAGTCATTCTGAAATAGTGTATGCGGCGACCGAGTTGCTCTTGCCTGGCGTCAACACGGGATAATACCGCGCCACATAGCAGGGCTTTAAAGTGCTCATCATTGGAAAACGTTCTTCGGACGAAAACTCTCAGGATCTTACCGCTGTTGAGGATCCCATTTCGATGCCAACCCACTCATTGCGCAACTGATCTTCAGCATCACTTTACTTTCACCAGCACGTTTTCTGGGTGAGCAAAACAGGAAGGCAAATGCCGCAAAAAGGGAATAAGGGCGACACGGAAATGTTGAATACTCATGCTCACTTCCTTTTTCAATATTATTGAAGCATTTATCAGGGTTATTGTCATGAGCGGATACATATATTTGAATGTATTTAGAAAATAAACAAATAGGTTCCGCGCACATTTCCCCGAAAGTGCCACCCTGACGTCTAAGAAACCATTATTATCATGACATTAACCTATAAAATAGGCGTACGGGCCCTTTTCGTCTTCAAACAAAAAATTC**CCATGGAGTCAAAGATTCAAATAGAGGACCTAACAAGAACTCGCCATGAAGACTGGCGAACAGTTCATCACAAGAGTCCCTTACGACTCAATGACAAGAAGAAAATCTTCGTCAACATGGTGGAAGACGACGCTTGTCTCAAAATATCAAGATACAGTCTCAGAGACCAAAGGGAACGGTGAGACTTCAACAAGGGTAATATCCGGAAACCTCCTCAGTTCCATTGCCCAGCTATCTGTCACTTTATTGTGAAGATAGTGGAAAAGAAGGTGGCTCCTACAAACCATCATTGCGATAAGGAAAGGCCATCGTTGAAGATGCCCCTCTGCCGACAGTGAGTCCCAAAGATGGACCCCACCCACGAGGAGCATCGTGGAAAGAAGACGTTCCAACCACGTCTTCAAGCAAGTGGATTGATGTGA** | 1109 bp |
| **14** | ccggggaacaatcgacaaaggtttgttttattcgcgaatccaacttgccattaattggctatgCAGAAGCGGCAAGTTACACGCAGGTTATCAATTATGGTTATGGCAGCACTGCATACCTCTTCTTACTGTCATGCCATCCGTAAGATGTTTCTGTGACTGGTGAGTACTCAACCAAGTCATTCTTTTGAGAATAGTGTATACGGCGACCGAGCGCCGTTTTGCCCGGCGTCAACACGGGATAATACCGCGCCACATAGTCCTTTAAAAGTGCTCATCATTGGAAAAACGTTCTTCGGGGCGAAAACTCTCAAGGATCTTACCGCTGTTGAGATCCAGTTGATGTAACCCCACCTCGTGCACCCAACTGGATCTTCAGCATCTTTACTTTCACCAGCGTTTCTGGGTGAGCAAAACAGAAGGCAAAATGCCGCAAAAGGGAATATATGACACGGAAATGTTGAATACTCATACTCTTCCTTTCAATATTATTGAAGCATTTTATCAGGGTTATTGTCTCATGAGCGGATACATATTTGAATGTATTTAGAAAATAAACAAATAGTTCCGCGCACATTTCCCCGAAAAGTGCCAACGGACGTCTAAGAACCATTAAGTTATCATGACATTAACCTATATAAAATAGGCGTATCACGAGGCCCTTCAAGAATT**CCATGGAGTCAAAGATTCAAACAGAGACCTAAACAGAACTCGCCGTAAAGACTGGCGAACAGTTCATACAGAGTCTCTACGACTCAATGACAAGAAGAAAAGTCTTCTTCGTCAACATGGTGGAGCACGACACGCTTGTTCACTCCAAAATATCAAAGATAACAGTCTCAGAAGACCAAAGGCAATTGAGACTTTTCAACAAAGGGTAATATCCGAAACCTCCTCGGATTTATCAGTTCAGCTATCTGTCACTTTATTGTGAAGATAGCTGGAA** | 946 bp |
| **15*** | CATCATTGGAAAACGTTCTTCGGGCGAAAACTCTCAGGGATCTTACTGCTGTTGGAATCAAAGTTCGATGTAAACCTACCTCGCACCCAACTGATCTTCAGCATCTTTACTTTCACCAGCGTGCTTCTGGGTGAGCAAAACAAGAAGCGTAAAATGCCGCAAAAAAGGAATAAGGAAGACACGGAAATGTTGAATACCATATTCTTTCCTTTTCAATATTATTGAAGCATTTATCAGGGTTATTGTCTCATGAGCGGATACATATTTGAAATGTATTTAGAAAATAAACAAATAGTTCCGCACATTTCCCCGAAAAGTGCCACCTGACGTCTAAGAAACCATTATTATCAAGTATATTAACTTATGTGGCGTATCACGAGGCCCCTTCGTCCCCAAGACCC**CCATGGAGTCAAAGATTCAAATAGAGGACCTAACAGAACTCGCCGTGTGATTCTGGCGAACAGTTCGCGTACAGAGTCTCTTACGACTCATCAATGAATAAGAAGAAATCTTCGTCGCAACATGGTGGAGCACGACAATACGCTTGTTTACTCCAAAATATCAAAGATACAGTCTCAGAAGAACCAAAGGGCAATTGAGACTTTTCAACAAAGGGTAATATCCGAAACTCCTCGATTCCACGCCGTCTAAAGTTATCTGGGTCACTTCATTGTGAAGATAGTGGAAAAGGAAGGTGGGCTCCTACAAATGCCATCATTAAGTAGATAAAGGAAAGGCCATCGCTTGAAGATGCTCGCCGACAGTGGTCCCAAAGATGGACCCCCACCCACGAGGAGCATCAGGAAAAAATAGCATGCTCCAAACCACGCCTCAAAGCAAGTGGATTGATGTGATAT** | 857 bp |
| **16** | tacctccggaacaatcgaaacaaggtttgaacattccagagtgaatccaacttgccgccactaattggttgttaaccGAAGTAAGTTGTACAGTGTTATCACTCGTATGGTTATGGCAGCACTGCATAATTCTCTCTTACTATCATACCATCAATGCGTGCTTTTCTGGGCCCCGAGGTGAGTGCTCCTTCAACCAGTCATTCTGAGGTATAGTGGCAATGCACGTGACACATCCGAGTTGCCTCTTGCCCAGCGTCAACTTACCGGGATGAAAATACCACCATAGCAGAACTTTAAAGATGCTTTCATCATTGGAAAACTTCTTCGAGACGAAAACTCTCCAAGGATCACCTGCTTCTTAATTTGGAGATCAGTTCGATGTAACCACTCTGTTCACGCCAACTGATCTTCGAAATATCTTTGCTTTTCCTGTAGCGTTTCACCAAGGGTGGAAACCTTAAAGCAGAGCATAAATCGCAAAAAGAATAGACGACGAATGTTGGAGAAATACATAATACTCTTCCTTTTTCAATATTATTGAAAGCATTTATCGAGGTTATTGTCGCCTTATTGGGCAGGATACATGTTGTTGGATGTATTATTTATGAAAAATAAACAAATGAGGGTTCCTACGCGCACATTTCCCCGAAAGTGCACAACCCTGACGTCTAGAAACCGGTATTATTATCCCTTGGTAGCATTAACTTGCTTTAAAATATGATAGGCGTATCACGAAGACACTGG**GACAGACGAACAGTTCGCGTACAAGTCTCTTACGATAATGACAAAGAAAATATCTTCGTCAACATGGTGGAGCACGTCACGCTTGTCTACTCAAAATATCAAAAGACTCAGAAGACCAAAGGGCAGTGAGACTTTCAGCAAGGTAATATCGGAAACTTCTCCTCTCCGGATTCATTGCCCAGCTATCTGTCACTTATTGTGAATAGTGGAAGCTCCTACAATGCCATCATGCGATAAAGGAAAGGCCATCGTTGAAGAGATGCACTACGCGACAATTGGTCCCCAAAGGTGGACCCCACCGAGACATCGTGGAAGAGACGAACAACCATTCTTTAAACAGGTGATGGATTGATGTGATA** | 1105 bp |
| **17** | cggggaacaatcgacatccatgaatccaacttcacaattaaattggttatgCAGAAGCAAGTTGGCCGCAGTTATCACTCATGGTTATGGCAGCACTGCATAATTCTCTTTACCGCCATCCGTAAGATGTGCTTTTCTGTGATCAGTAGAGTACCAACCAAGTCATTCGATGAATAGTGTATGCGTTAATTCCGAGTTGCTCTTGCCCGGTCAACACGGATAATACCGCGCCACATAGCAGAACTTAAAAGTGCTCTATCGAAAACGTTCTTCGTAGAAAACTCTCAAGGATCTTTACCGTTGAGATCCAGTTCGATGTAACCCACTCGTGCACCCAACTGATCTTCAGCATCTTTACTTTCACCAGCGTTTCTGGGTGAGCAAAACAGGAAGGCAAATGCCGCAAAAAGGGAATAGCGACACGGAAATGTTGAATTATTACCTTCCCCTTTTTCAATATTATTGAAGCATTTATCAGGGTTATCATCTCAAGAGCGATACATATATCGAATGTATTTAGAAAATAAAACAAATAGGGGTTCCGCACATTTCCCTAGAAAAGTGCCACCTACCTGCCTACAGAACCATTATTATCATGACATTAACCTATAAAAATAGGCGTACGAGGCCCTTCGTCTTCAAGAATTC**CCATGGAGTCAAAGATTCAAATAGAGGACCTAACAGAAACTCGCCGTAAAAGATGGCGGAACGTTCATACAGAGTCTCATGACTCAATGACAAGAAGAAAATTGTCAACATGGTGAGCACTTATCATTGTCTACTCCAAAATATCAAAAGATACAGTCTCAGAAGACCAGCAGCAATTGAGACTTTTTCAACGTAAAGGGTAATATCCGAAACCTCTCGATTCCTGAGTGAGCTGCCCAGCATTATCTGTCACTTATTGAAGATAGTGGAAAGGAAGGTGGCTCCTACAAATGCCATCATTGGTGATAAAGGAAAGGCCATCGTTGAAGATGCTCTCGCCGACAGTGAACCCAAAGATACCACCACCACGAGGAGAGCCGAAAAAGAAGATGCCCAAACCATCGCCCAAAGCAA** | 1062 bp |
| **18** | caattggggcattaatgtatctttactaatgtaccgacagacattgcttttctgtaacaatagcagatttagttcttccccgacacaaagacactggaatggtatcaaacatttgaccctgaattacctccxaggaacaatcgataaaggtttgtttattcaagagtgaatccaacttgcccactgagttggttatgCAGAAGTAAGTTGTACAGATGTTATCACTCATGGTTATGGCTTGTACTGCATAAATTCTCTTGCCCCTGTCATGCCATCATCAAGCAAGATGCTGCAATGACTGGTGATGCTCAACTTAAGTCATTCTGAGAATAATTGTCGACGACCAGGTTGCTTTCTTGCCCGGCGATCATCACGGGATAATACCGCGCCACATAGCAGAACTTTAAAGGGTGCTCATCATTGGAAGAAACGCGTTCTTTCGGGGCGAAAACTCTCGGGATCCTTACCGCTGTTGAGATCCAGTTCGATGTAACCTCGTGCACCCAACTGATCTTCCAGCATCTTTTACTATCACCAACGTTTCTAGATTTGAGAGCCTTTGAAAACAGGAAGGCAAAATGCACCAAAAGGGGAATAAGGAGCATTACGGAAATGTTGAATATAATACTCTTCCTTTTCAATATTATTGAAGCATTTATCAGGGTTATTGTCTCATGAGCGGATACATATTTGATGTATTTAGAAGAAATATAAGCAAAACAGGGTTCCTGCGCACATTTCGAAAGCGCCACCTGATCTAAGAAACCATTATTATCCATGACATTAACCACAAAATAGGCATTATCACGAGGCCTTTCGTCTTCAAGAATTC**CCATGGAGTCAAAGATTCAAATAGAGGACCTAACAATTTCGCCGTAAAGAGACTGGCGAACAGTCCCATACAAGTCTCTTACGACTCAATGACAAGGAAAGAAAATCTTCGTCAACAT** | 950 bp |
| **21** | ccttcagatatcttataagtctggcgtgcgtgaaaacttgatatgcgtctgatggatgttgtcactgcctatttatatggctcgttggacaacgaaatatttatgaaaatccctgaaggattcaaaatgcctgaagcatttaaaggttcccgagaaacttgttcaataaagcttcataaatccttatacggattgaaacaatccggtcgtatgtggtacaaccatagcagtaccactactgaaagaagggtataaaatgatccagtttgtcgttgtgtctttataaagggtctggatctgagtttgttataattgctgtgtatgttgatgacttgaatattattggaactccacagagattccaaaggcagtagactatttgaagaaagaatttgaaatgaaagatcttggaaagacaaattttgtcttggtcttcaaattgagcatttgataaatggaattttatccatcaatcaacatacactgaaaagattttaaagattttatatggataaatctcatccattgagtactccgatggtcgtgagatcgcttgacataaataaagatccatttcgccctcatgaaaatgatgaaagattcttggtgctgaaacaccatatcttagtgcaattgggcattaatgtatcttgctaatacccgaccagacattgctttttctgtaaacttattagcaagatttagttcttccccgacacaaagacactggaatggtatcaaacatttattcagatacctccgaggaacaatcgacaaaggtttgttttattcaagtgaatccaacttgccactaattggttatgCAGAAGTAAGTTGGCCAGTGTTATCACTCATGGTTATGGCAGCACTGCATAATTCTCTTACTGTCATGCCATCCGTAAGATGCTTTTCTGTGACTGGTGAGTACTCAACCAAGTCATTCTGAGAATAGTGTATGCGGCGACCAGTTGCTCTTGCCCGGCGTCAACACGGGATAATACCGCGCCACATAGCAGAACTTTAAAAGTGCTCATCATTGGAAAACGTTCTTCGGGGCGAAAACTCTCAAGGATCTTACCGCTGTTGAGATCCAGTTCGATGTAACCCACTCGTGCACCCAACTGATCTTCAGCATCTTTTACTTTCACCAGCGTTTCTGGGTGAGCAAAAACAGGAAGGCAAAATGCCGCAAAAAGGGAATAAGGGCGACACGGAAATGTTGAATACTCATACTCTTCCTTTTCAATATTATTGAAGCATTTATCAGGGTTATTGTCTCATGAGCGGATACATATTTGAATGTATTTAGAAAAATAAACAAATAGGGGTTCCGCGCACATTTCCCCGAAAGAGAGCCACCTGACGTCTAAGAAACCATTATCATGACATTAACCTATAAAAATAGGCGTATCACGAGGCCCTTTCGTCTTCAAAATTC**CCATGGAGTCAAAGATTCAAATAGAGGACCTAACAGAACTCGCCGTAAAGACTGGCGAACAGTTCATACAGAGTCTCTTACGACTCAATGACAAGAAGAAAATCTTCGTCAACATGGTGGAGCACGACACGCTTGTCTACTCAAAATATCAAAGATACAGTCTCAGAAGACCAAAGCAATTGAGACTTTTCAACAAAGGGTAATATCCGAAACCTCCTCGGATTCCATTGCCCAGCTATCTGTCACTTTATTGTGAAGATAGTGGAAAAGGAAGGTGGCTCCTACAAATGCCATCATTGCGATAAAGGAAAGGCCATCGTTGAAGATGCCTCTGCCGACAGTGGTCCCAAAGATGGACCCCACCCACGAGGAGCATCGTGGAAAAGAAGACGTTCCAACCA** | 1839 bp |
| **22** | accttcagatatcttatataagtctggcagtgcgtgaaaaacttgatatgcgtctgatggatgttgtcactgcaccctattgctatatggctcgttggacaacgaagtatttatgaaaatccctgagttcaaaatgccctgaagcatttaaggttcgaaacttgttcaataaaacttctataaatcgccgccacagttgaaacaatcggtcgtatgtggtacaaccatagcgagtacctactgaaagaagggtatagaaatagtcagtttgtcgttgtgtctttataaaggttaccggatctggagtttgttatattgctgtgtatgttgatgacttgaatattgatacttggaactcacagagattccaaaggcagtagactgagaagaagaatttgaaatgaaagatcttggaaaatttttgtcttggtcttcaaattgagcattttgataatcggaatttttatcaatcaatcaacatacactgaaagattttaaagagattttatatggataaatctcatccattgaaattactccgatggtcgtgagatcgcttgacataaataaagatcatttcgccctctcatgaaaatgatgaaaagattcttggtgctgaaacaccatatcttagtgcaattggggcattaatgtatcttgctaatagtaccaaaccagacattgcttttcagtaaacttgtgagatttggttcttccccgacacaagacctggaatggtatcagaaacatttattcagatacctccaggxaacaatcgacaaggttttattcaaattatgaatcaacttgccataattggttatgcGAAGCGAAGTTGGCTGGTGTTATCACTCATGGTTATGGCAGCACTGCATAATTCTCTTACTGTCATGCCATCCGTAAGATGCTTTTCTGTGACTGGTGGTACTCAACCAAGTCATTCTACGAGAATAGTGTATGCGGCGACCGAGTTGCTCTTGCCCGGCGTCAACACGGGATAATACCGCACATAGCAGAACTTTAAAAGTGCTCATCATTGGAAAACTTCTTCGGGGCAGCTCAAGGATCTTACCGCTGTTGAGATCCAGTTCGATGTAACCCACTCGTGCACCCAACTGATCCTTCAGCATCTTTTTACTTTCCACCAACGTTTCTGGGTGAGCAGAAACAGGAAGGCAAATACCGCAAAAAAGGGGAATAGGGCGACACGGAAATGTTGAATACTCCATACTCTTCCTTTTTCAATATTATTGAAGTATTTTATCAGGGTTATTGTCTCATGAGCGGATACATATTTGAATGTATTTAGAAAAATAAACAAATAGGGGTTCCGCACATTTCCCCGAAAGTGCCACCTGACGTCTAAGAAACCATTATTATCATGACATTAACCTATAAAAATAGGCGTATCACGAGGCCTTTTCGTCTTCAAGAATTC**CCATGGAGTCAAAGATTCAAATGAGGGACCTAACAGAACTCGCCGCAAGACTGGCAGACAGTTCATACAGAGTCTCTTACGACTCAATGACAAGAAAATCTTCGTCAACATGTTGGGCACGACACGCTTGTCCTACTCAAAAATATCAAAGATACAGTCTCCAAGAAGACCAGGGCAATTGAGACTTTTCAACAAGGGTAATATCCGGAAACCTCCTCGGATTCCATTGCCCAGCTATCTGTCTTTATTGTGAAGAT** | 1695 bp |
| **23** | ttgctgtatgttgatgacttgaatattattggaactcccacagagattccaaagcagtgaactatttgaagaaagaatttgaaatgaaagatcttggaaagacaaattttgtgtcttggtcttcaaattgagcatttgataaatggaattttatccatcaatcaacatacactgaaagattttaaagagattttatatggataaatctcatccattgagtactcgatggtcgtgagatcgcttgacataaataaagatccatttcgccctcatgaaaatgatgaaaagattcttggtgctgaaacaccatatcttagtgcaattggggcattaatgtatcttgctaataatacccgaccagacattgcttttctgtaaacttattagcaagatttagttcttccccgacacaaagacactggaatggtatcaaacatttattcagatacctccgaggaacaatcgacaaaggtttgttttattcaagtgaatccaacttgccactaattggttatgCAGAAGTAAGTTGGCCAGTGTTATCACTCATGGTTATGGCAGCACTGCATAATTCTCTTACTGTCATGCCATCCGTAAGATGCTTTTCTGTGACTGGTGAGTACTCAACCAAGTCATTCTGAGAATAGTGTATGCGGCGACCGAGTTGCTCTTGCCCGGCGTCAACACGGGATAATACCGCGCCACATAGCAGAACTTTAAAAGTGCTCATCATTGGAAAACGTTCTTCGGGGCGAAAACTCTCAAGGATCTTACCGCTGTTGAGATCCAGTTCGATGTAACCCACTCGTGCACCCAACTGATCTTCAGCATCTTTTACTTTCACCAGCGTTTCTGGGTGAGCAAAACAGGAAGGCAAAATGCCGCAAAAAGGGAATAAGGGCGACACGGAAATGTTGAATACTCATACTCTTCCTTTTCAATATTATTGAAGCATTTATCAGGGTTATTGTCTCATGAGCGGATACATATTTGAATGTATTTAGAAAAATAAACAAATAGGGTTCCGCGCACATTTCCCAAAAAGTGCCACCTGACGTCTAAGAAACCATTATTATCATGACATTAACCTATAAAATAGGCGTATCACGAGGCCCTTTCGTCTTCAAAATTC**CCATGGAGTCAAAGATTCAAATAGAGGACCTAACAGAACTCGCCGTAAAGACTGGCGAACAGTTCATACAGAGTCTCTTACGACTCAATGACAAGAAGAAAATCTTCA** | 1237 bp |

**Supplementary file 4:** **Alignment of sequences from the supplementary file 3 corresponding to the unnatural association of the p35S and *bla* elements.**

| **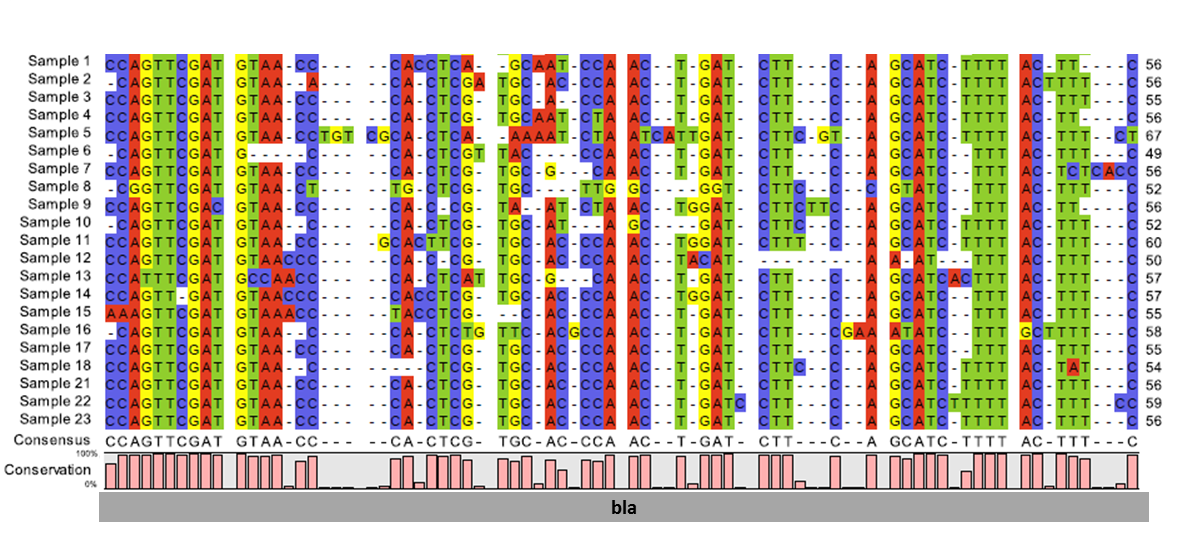** |
| --- |
| **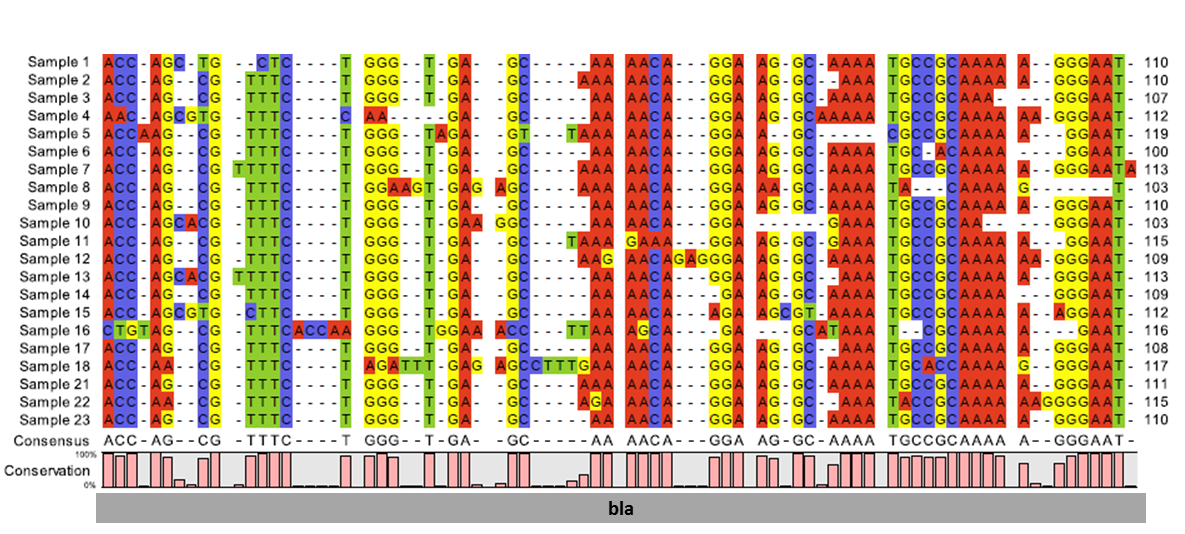** |
| **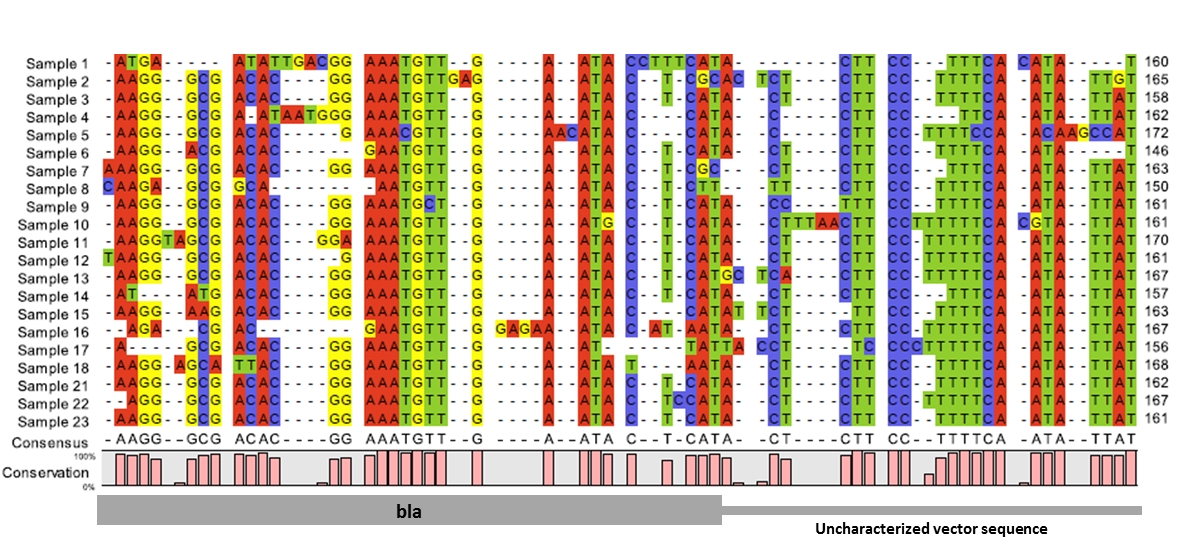** |
| **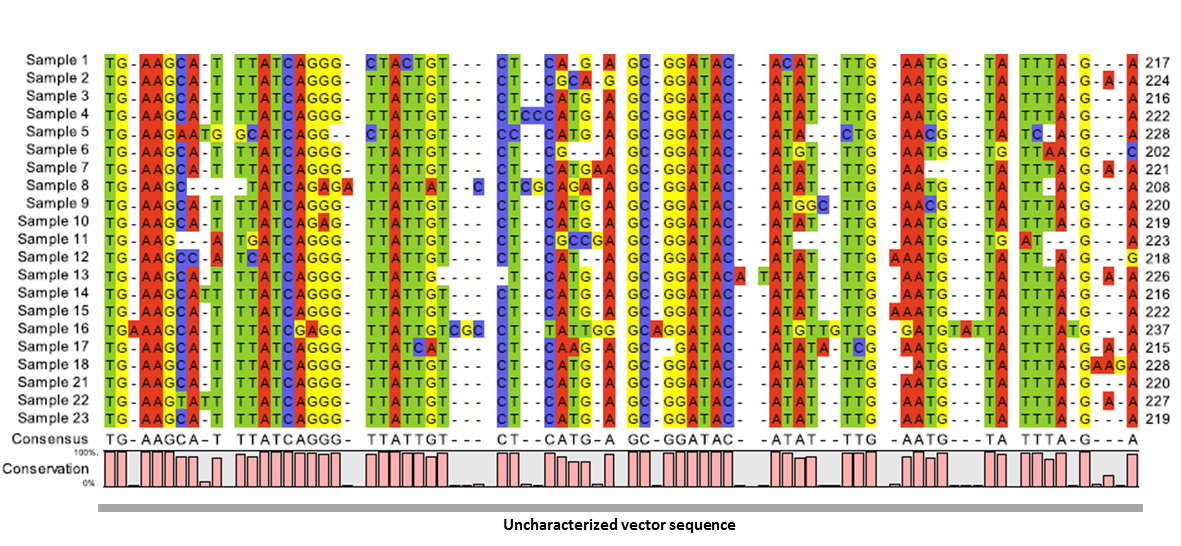** |
| **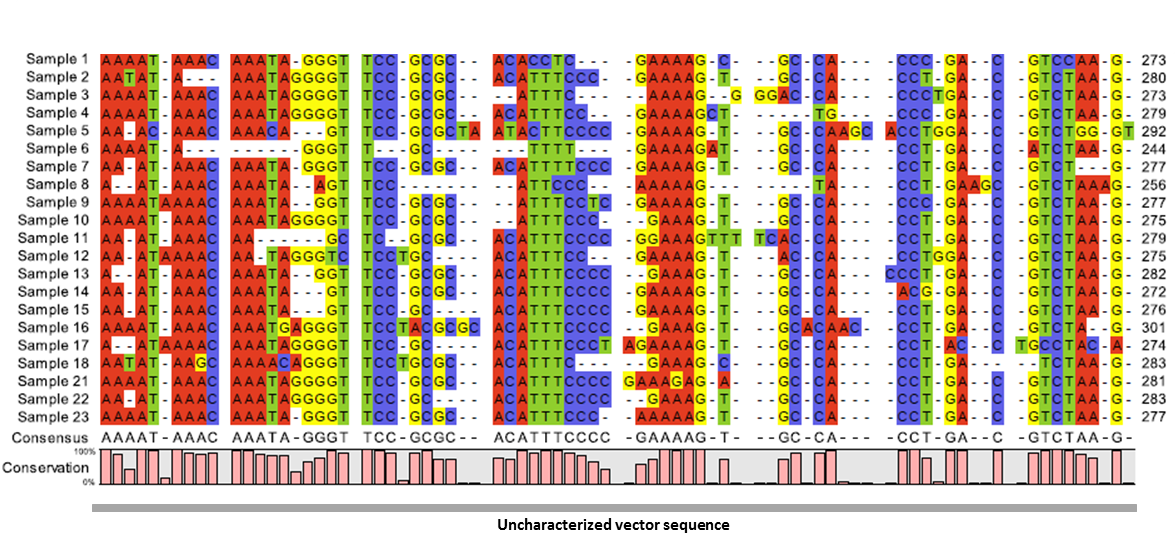** |
| **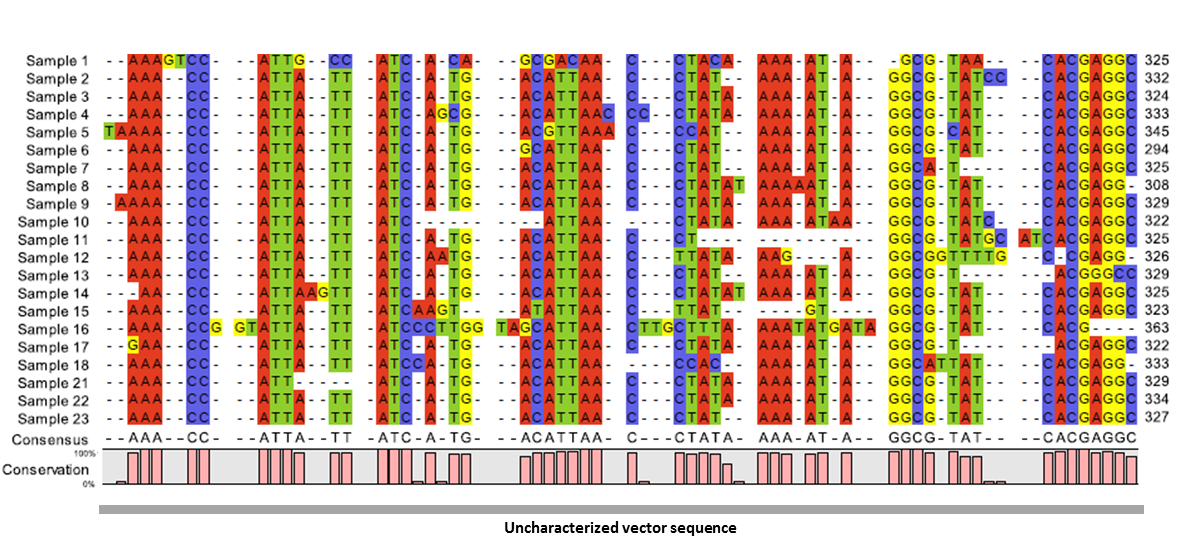** |
| **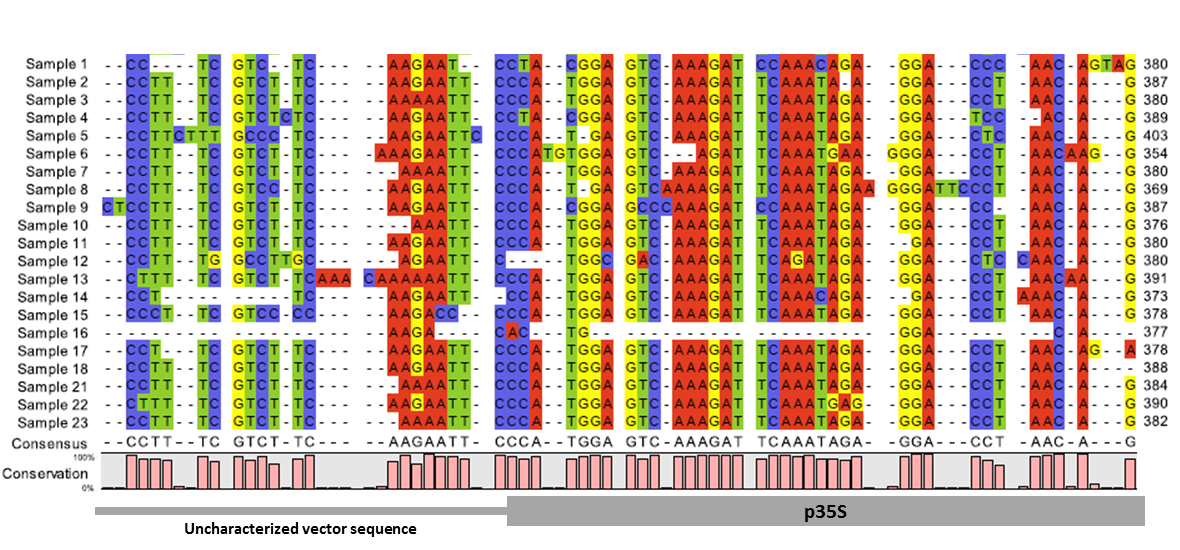** |
| **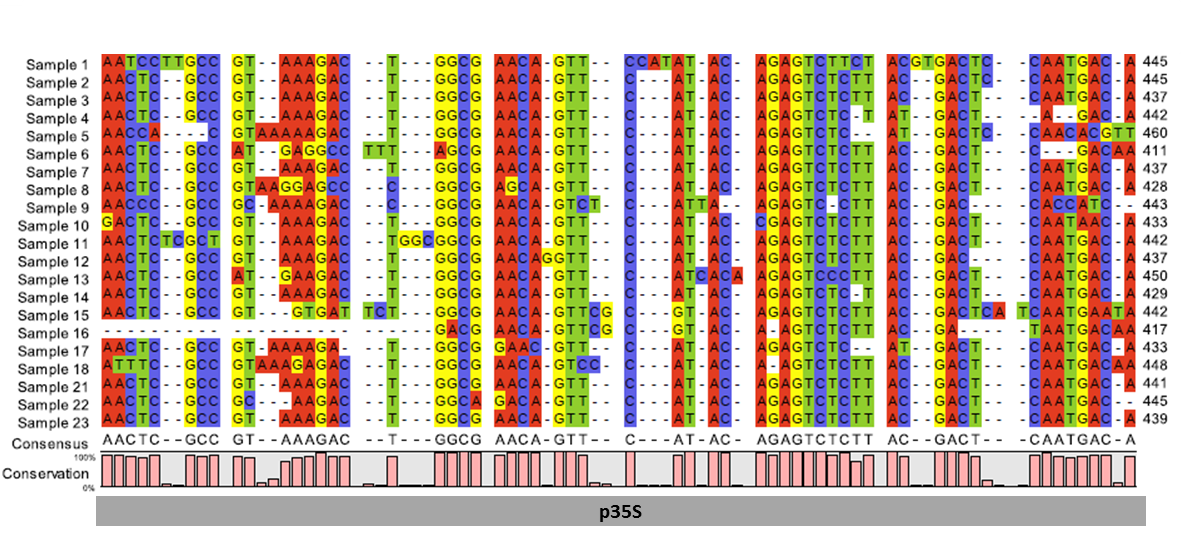** |
| **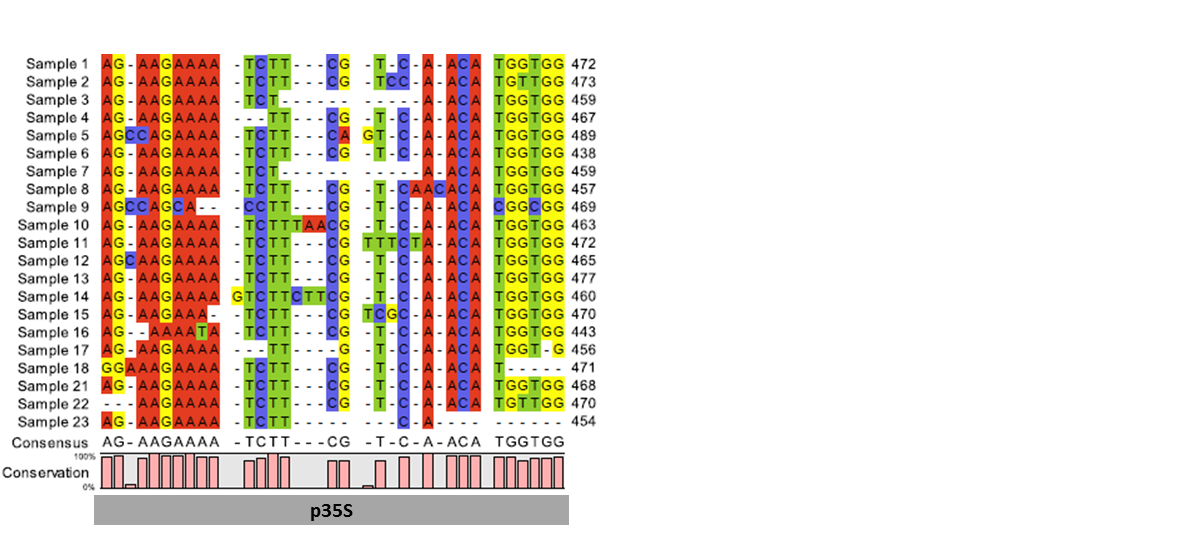** |

**Supplementary file 5: Verification by PCR of the transgene flanking regions identified on GM petunia samples (n°1-18 and n°21-23).**

(A) Visualization of the PCR products. The WT petunia samples (n°19-20) were use as negative control. The molecular-weight size marker is going from 15 to 10 000 bp.

(B) Alignment of sequences from the PCR products. The nucleotides corresponding to the petunia and the transgenic cassette are delimited by a black line.

| A |
| --- |
| 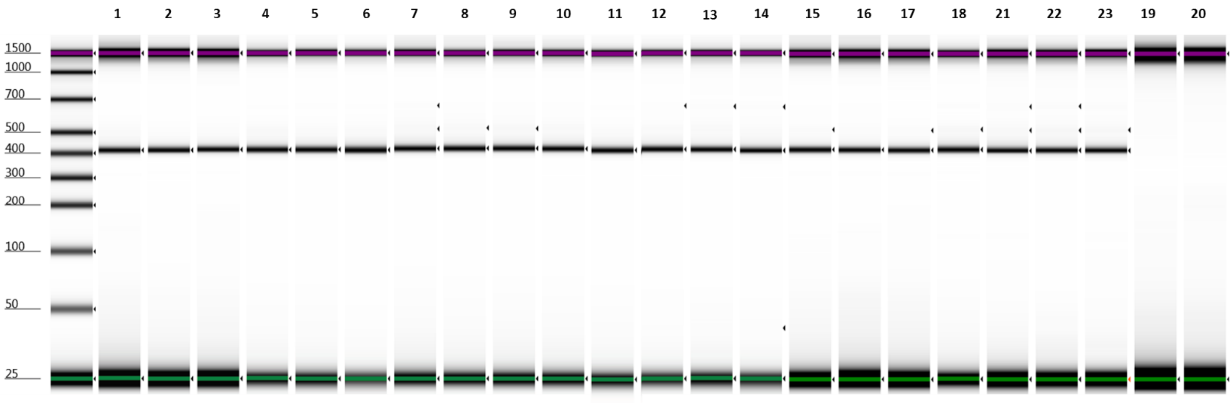 |
| B |
| 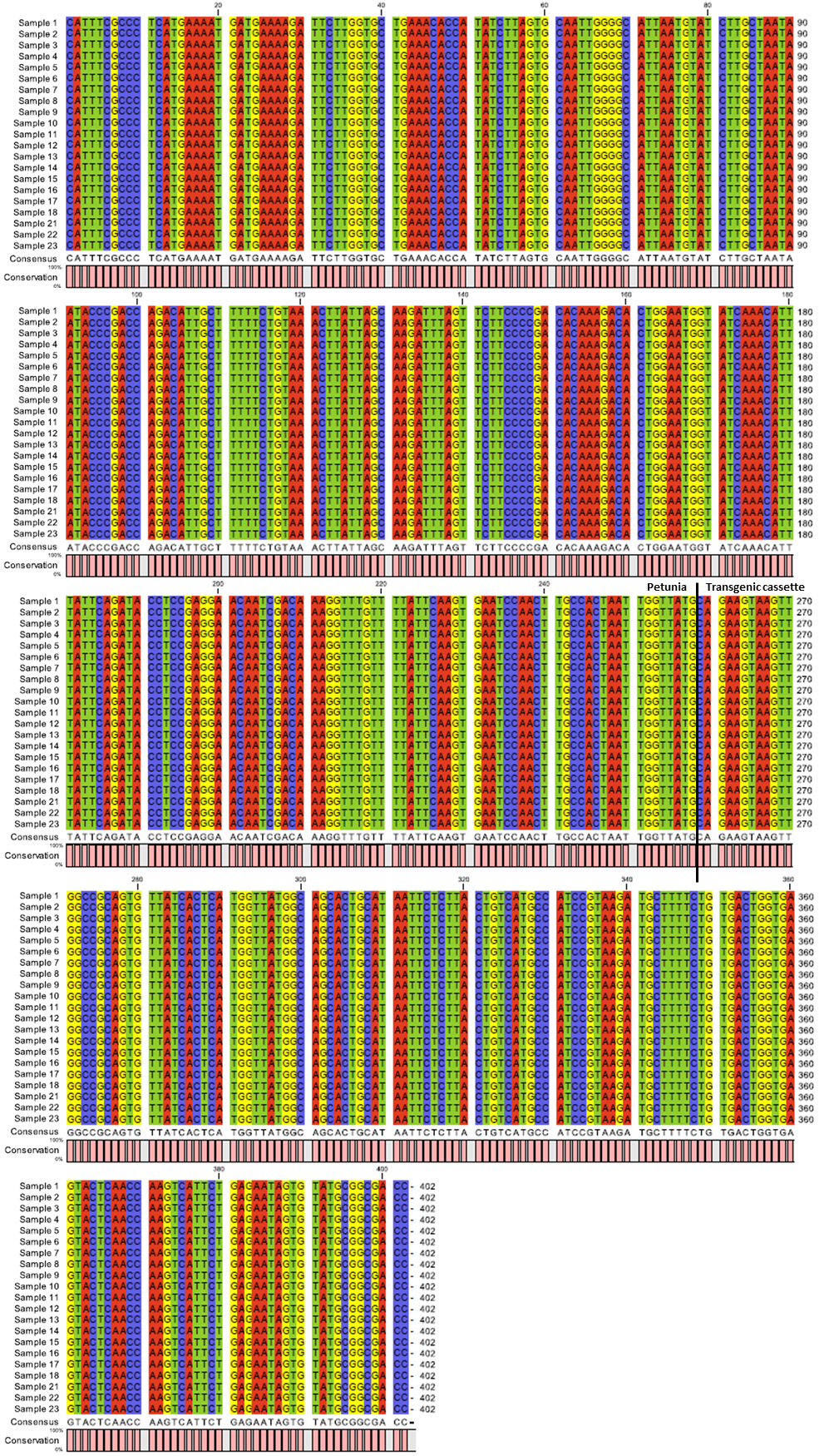 |

**Supplementary file 6:** **Comparison of the sequence of the transgene flanking region produced by the MinION and Sanger platforms.**

1. Alignment of the sequences of the transgene flanking region produced by the MinION (sample n°1-3, n°5, n°7-9, n°11-14, n°16-18 and n°21-23; supplementary file 3) and Sanger platforms (referred as “confirmed”; Supplementary file 5). The nucleotides corresponding to the petunia and the transgenic cassette are delimited by a black line. The sequences of the primers (Junction-F and Junction-R; Table 1) used to confirm the transgene flanking region are indicated.
2. Alignment of the consensus sequence of the transgene flanking region produced by the MinION (point A) and Sanger platforms (Supplementary file 5). The nucleotides corresponding to the petunia and the transgenic cassette are delimited by a black line.
3. Individual alignment of the sequences of the transgene flanking region produced by the MinION (sample n°1-3, n°5, n°7-9, n°11-14, n°16-18 and n°21-23; supplementary file 3) and the Sanger platforms (referred as “consensus Sanger”; Supplementary file 5). The nucleotides corresponding to the petunia and the transgenic cassette are delimited by a black line.

| **A** |
| --- |
| 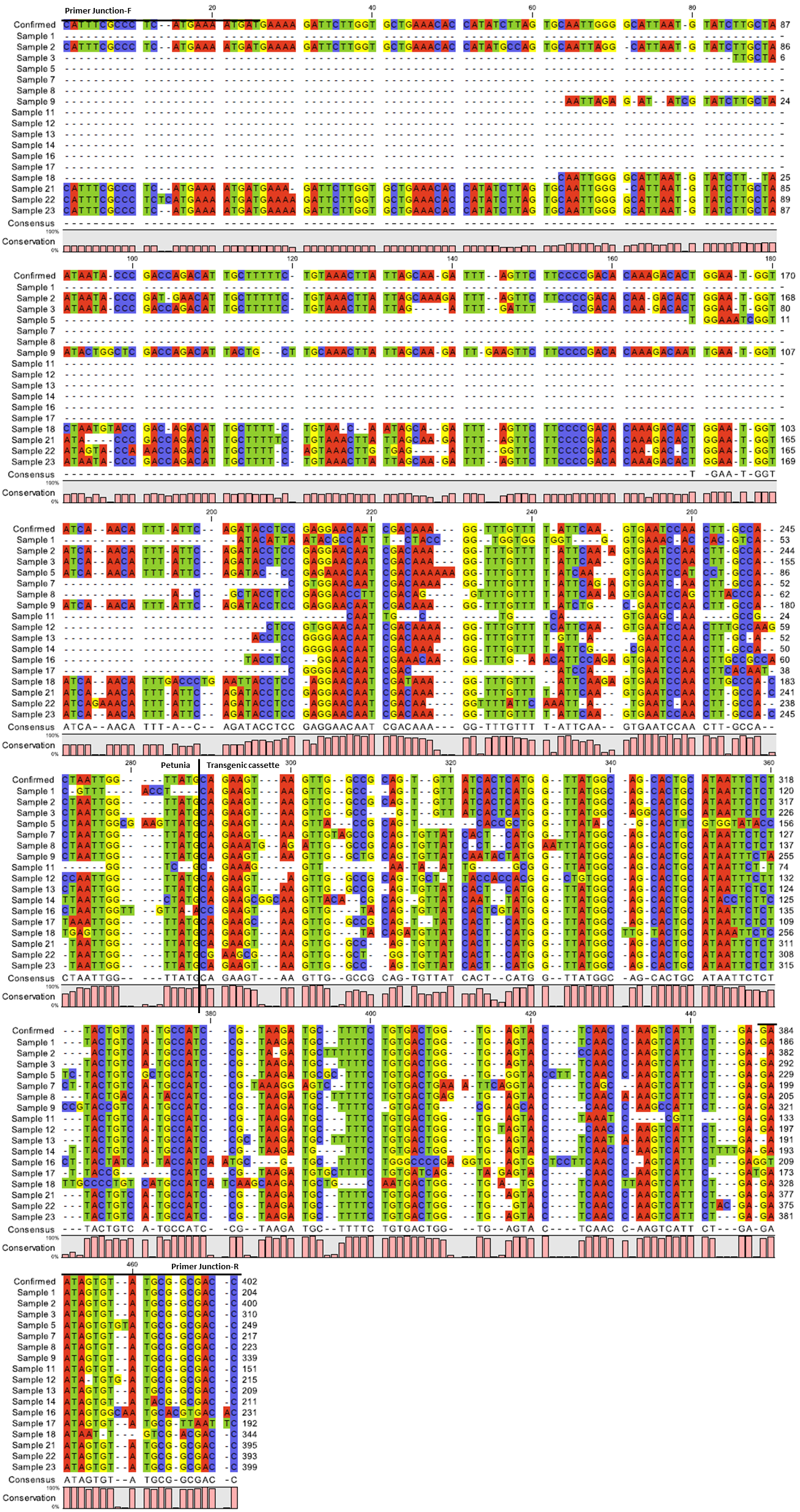 |
| **B** |
| 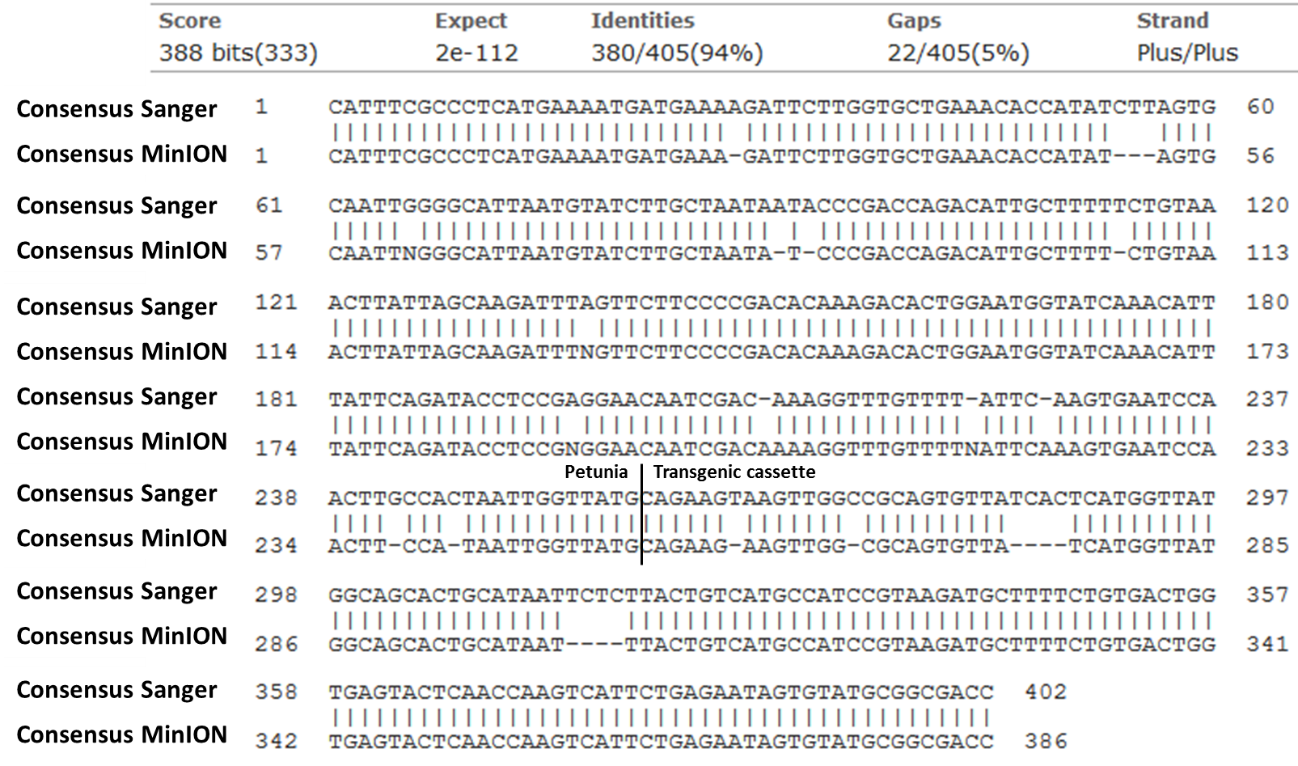 |
| **C** |
| 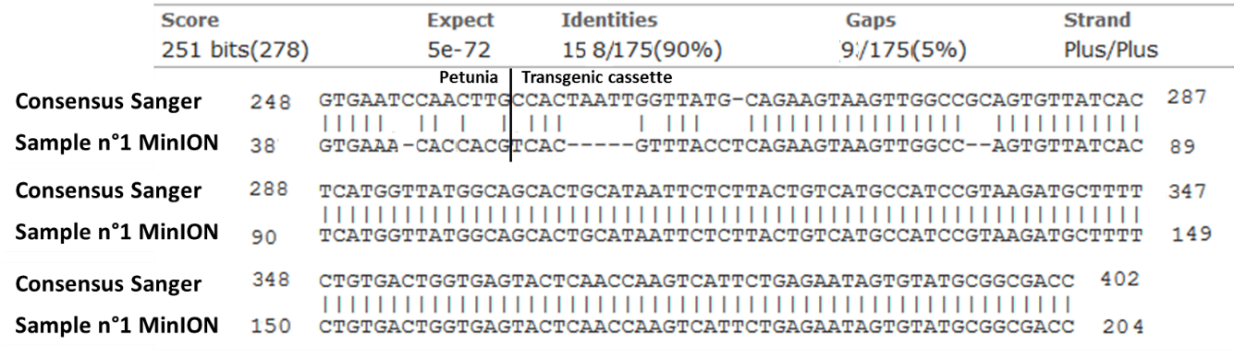 |
| 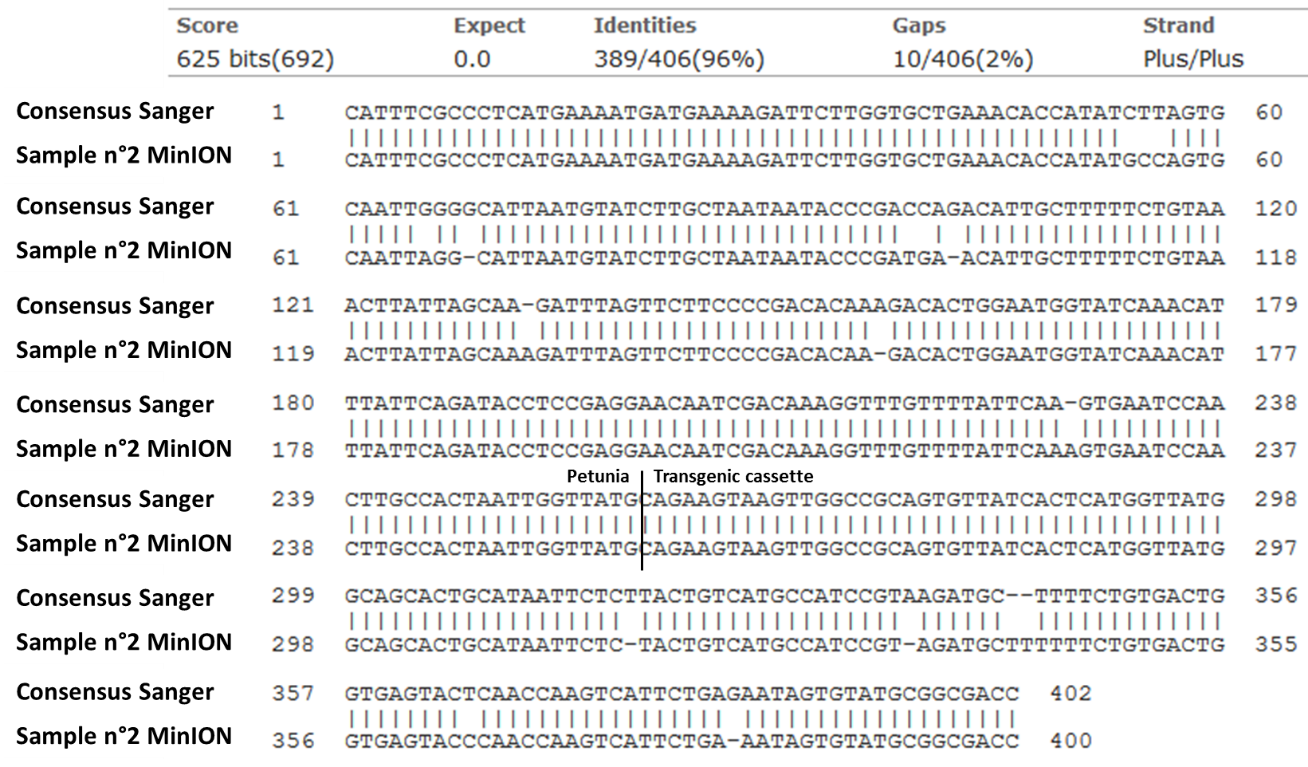 |
| 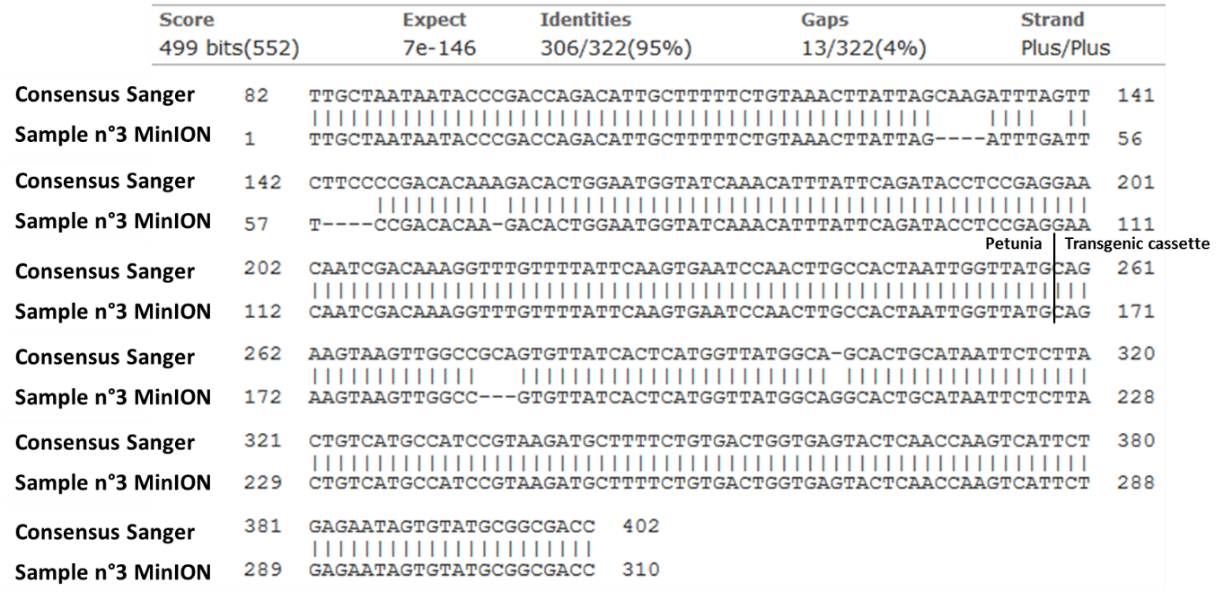 |
| 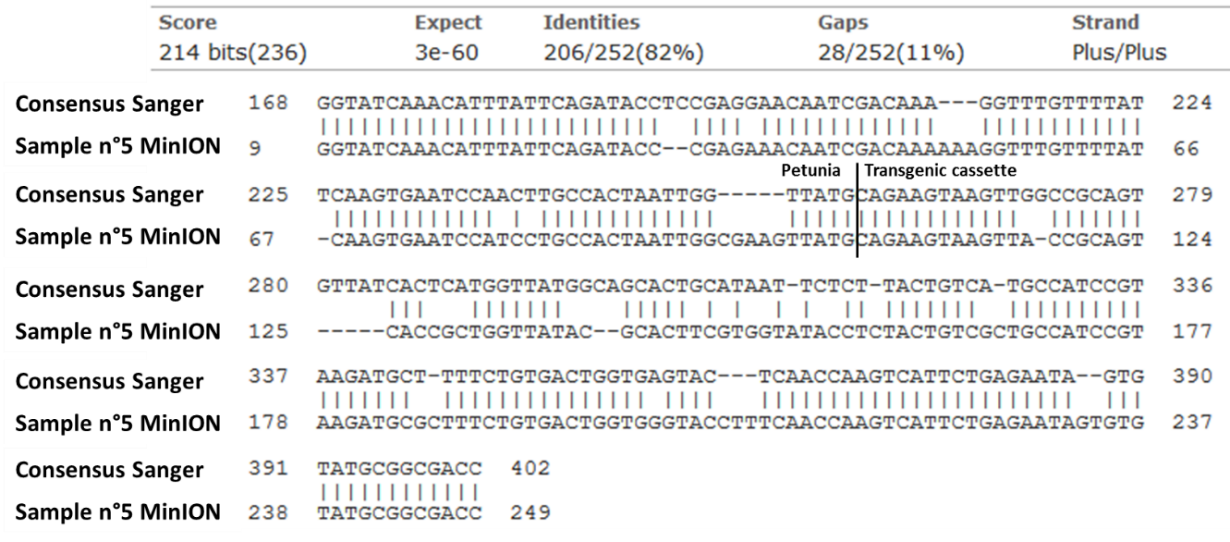 |
| 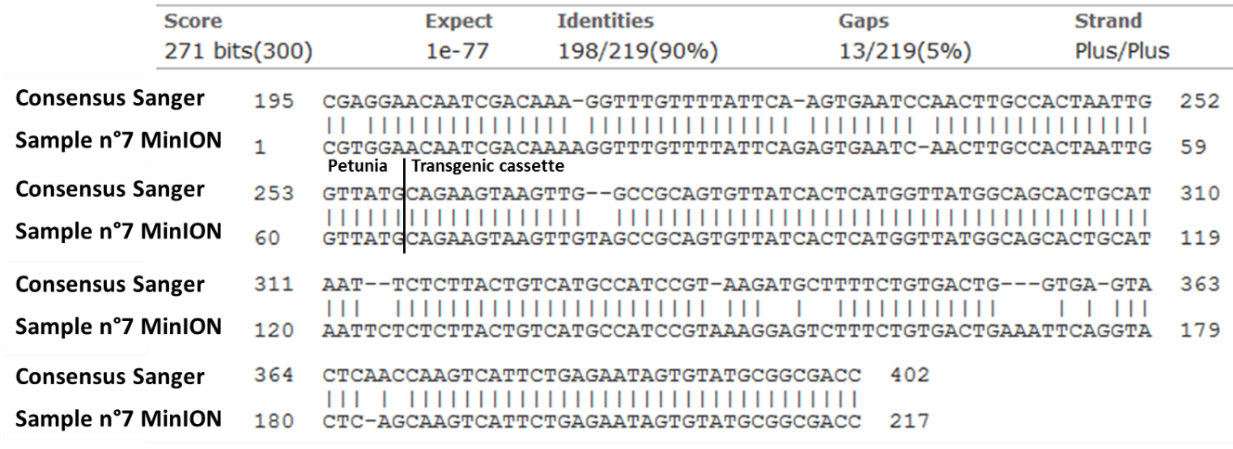 |
| 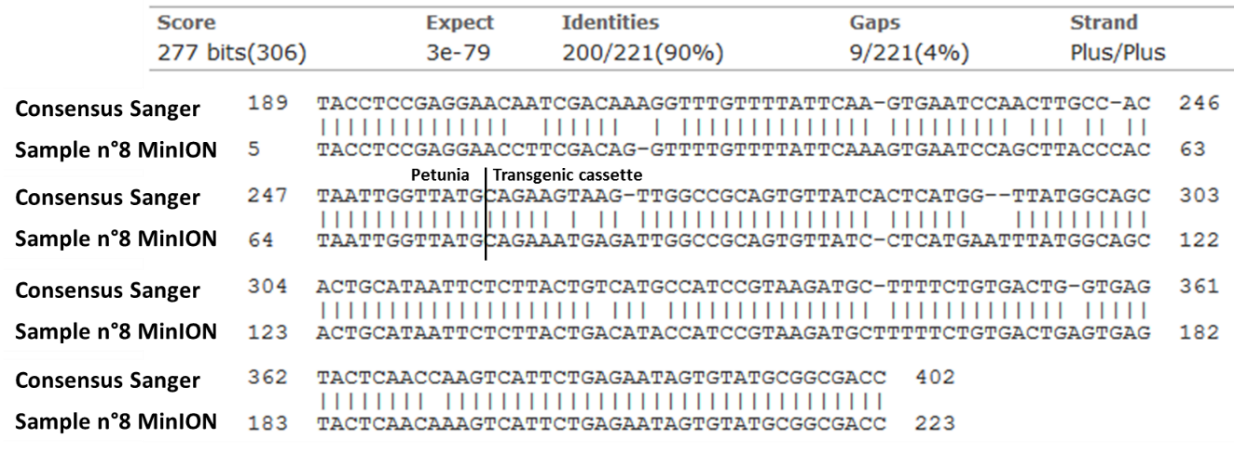 |
| 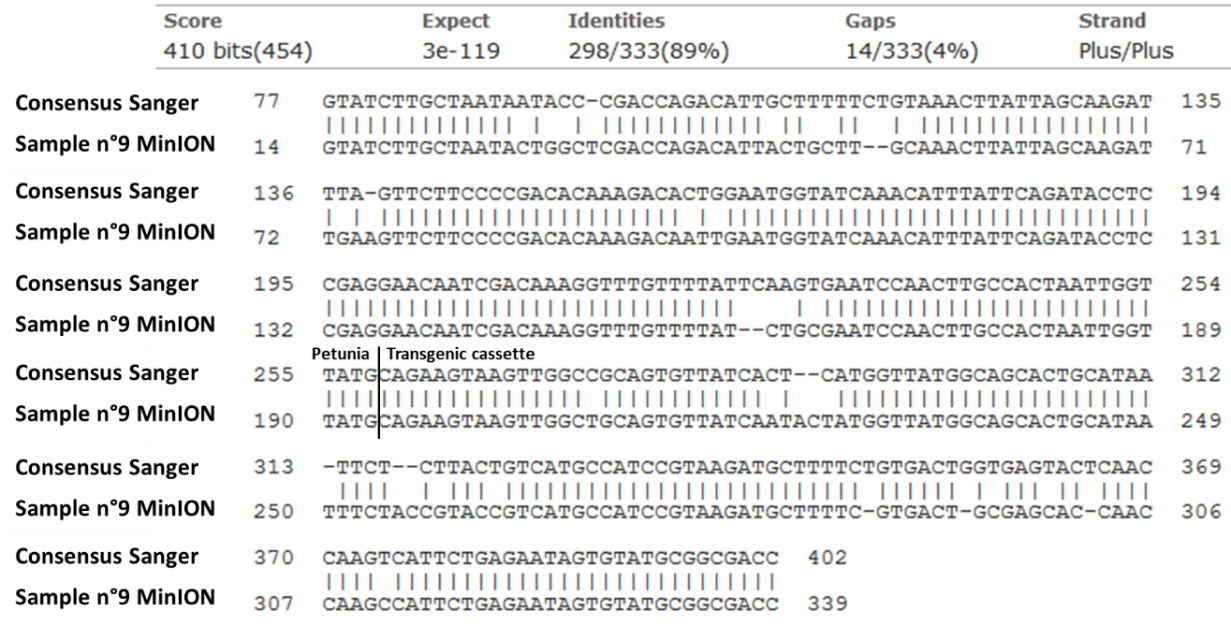 |
| 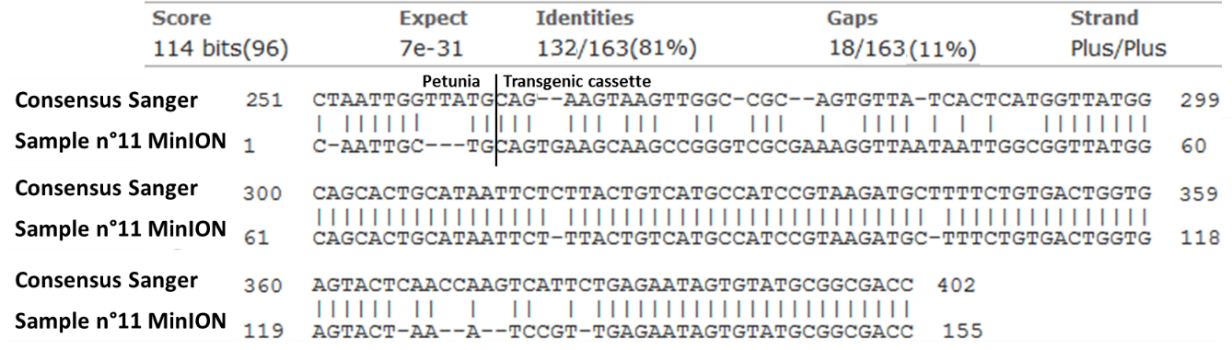 |
| 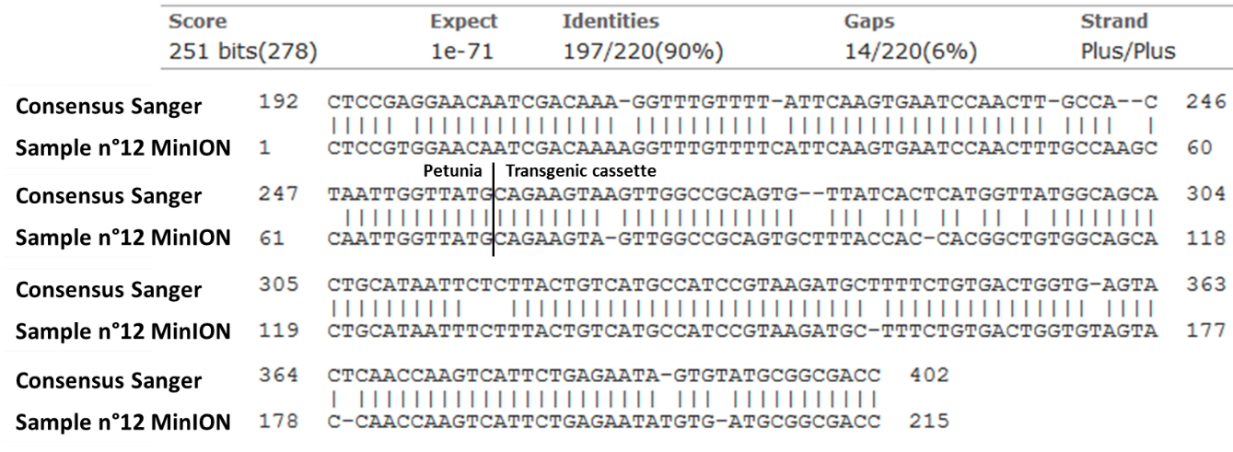 |
| 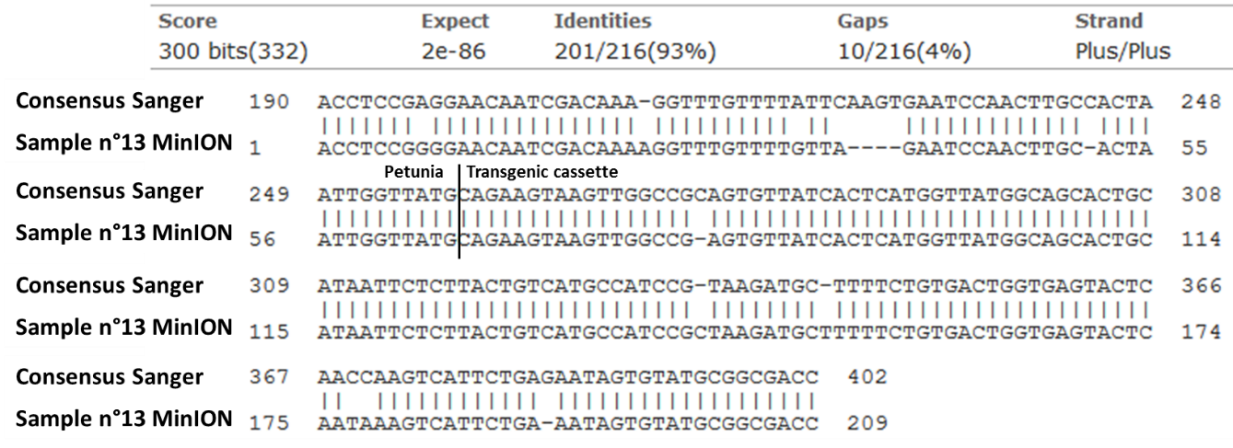 |
| 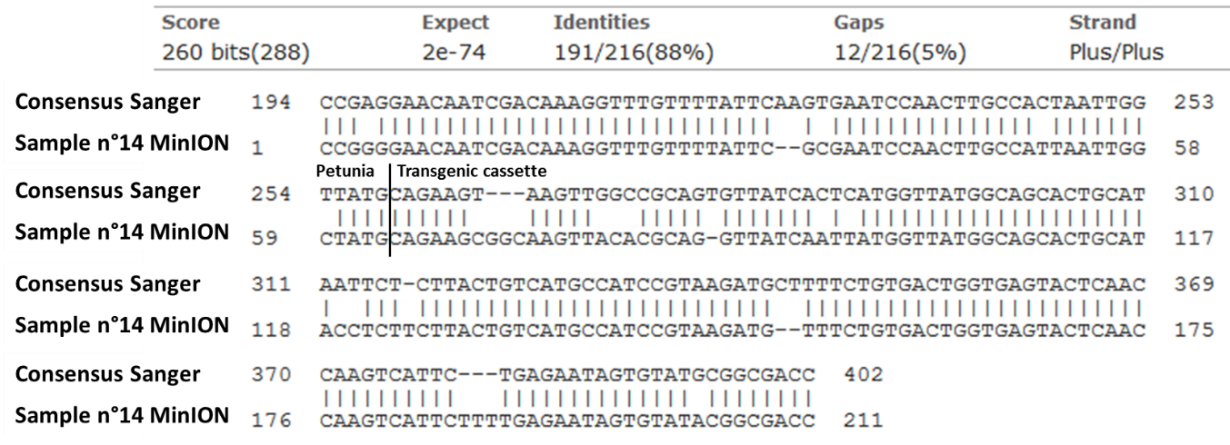 |
| 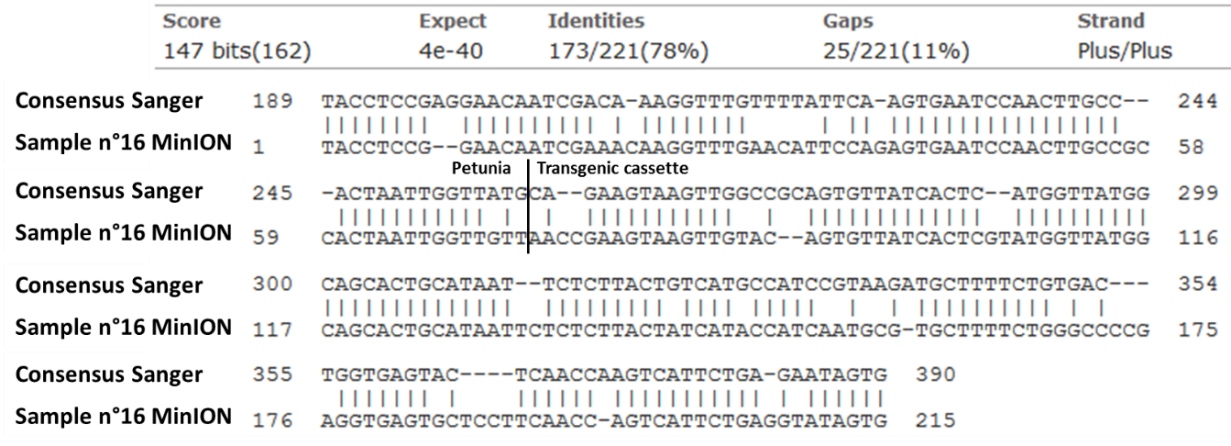 |
| 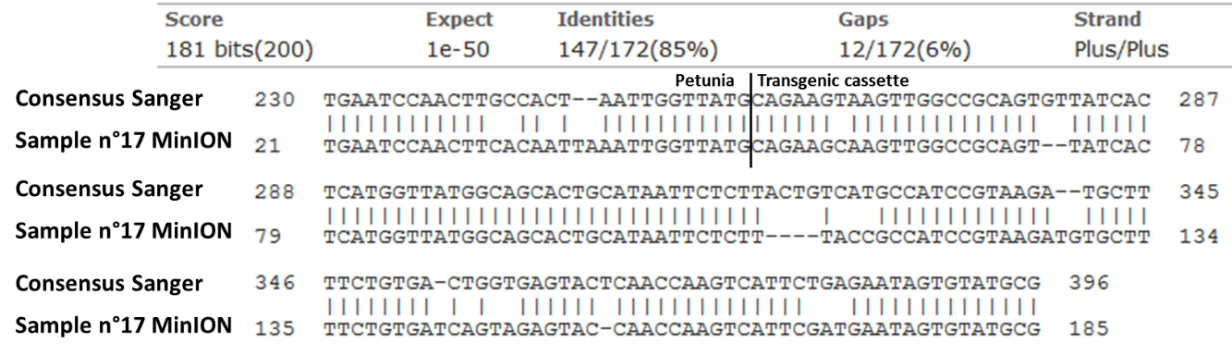 |
| 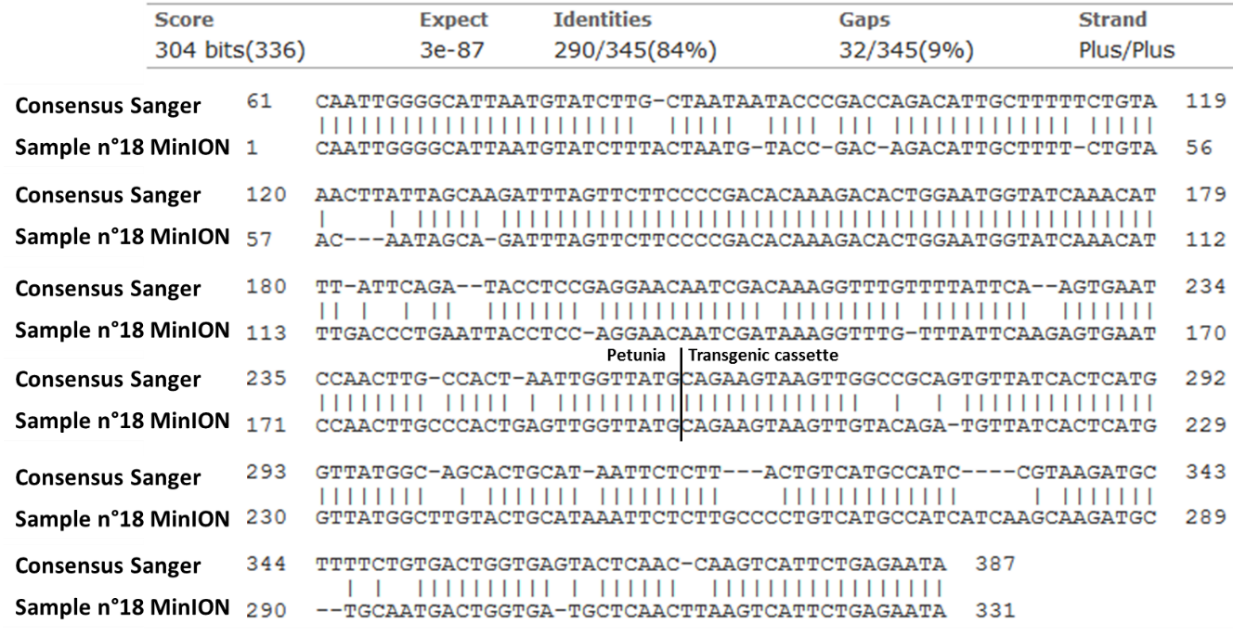 |
| 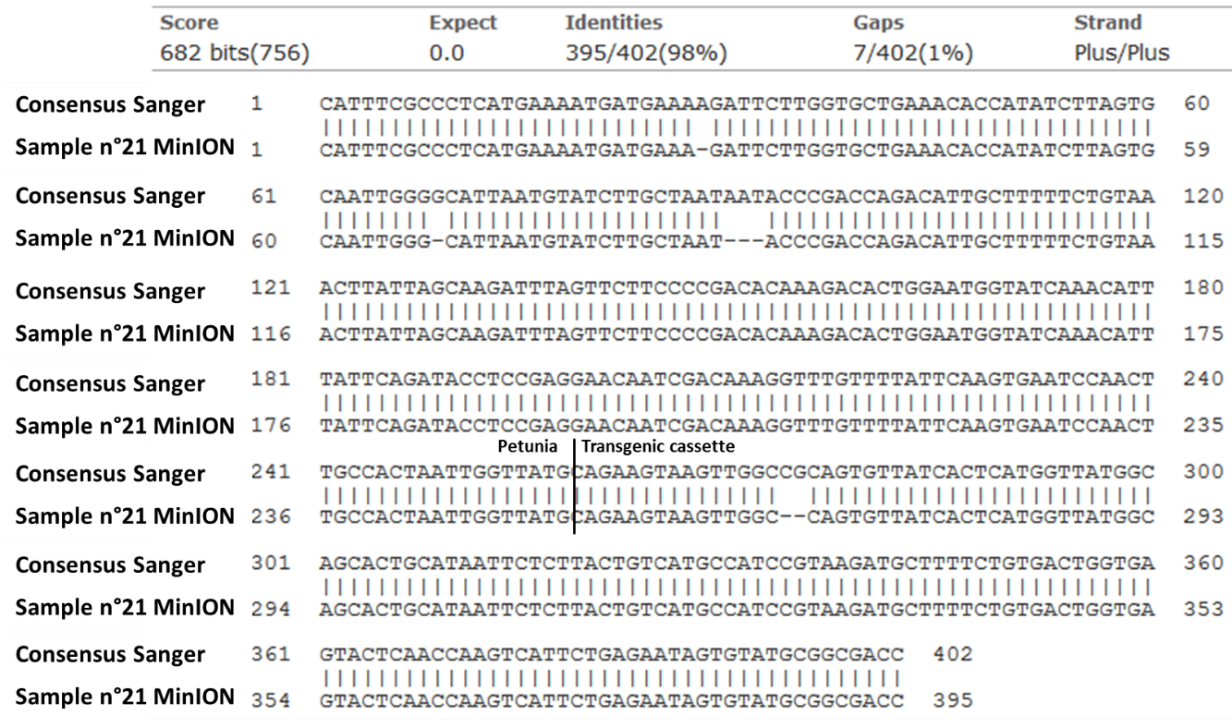 |
| 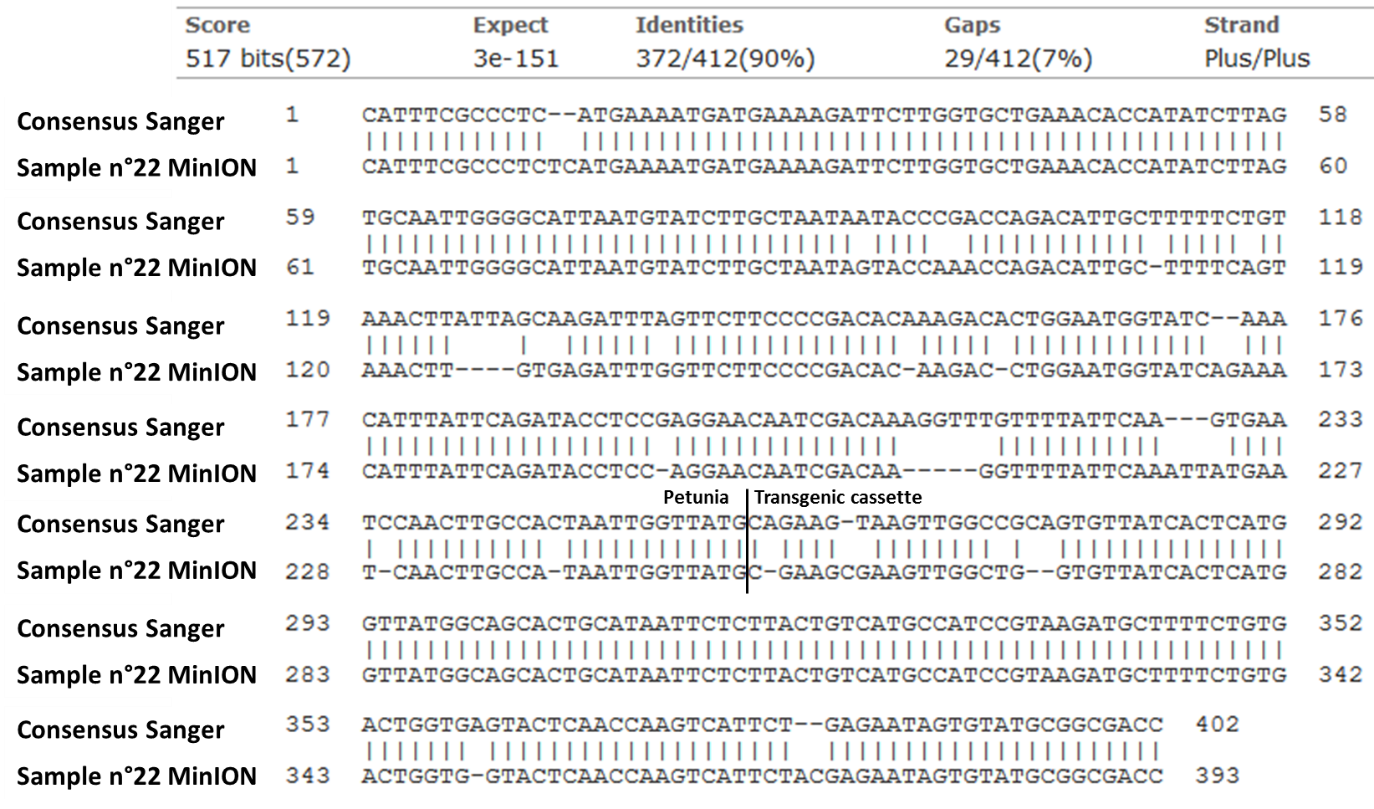 |
| 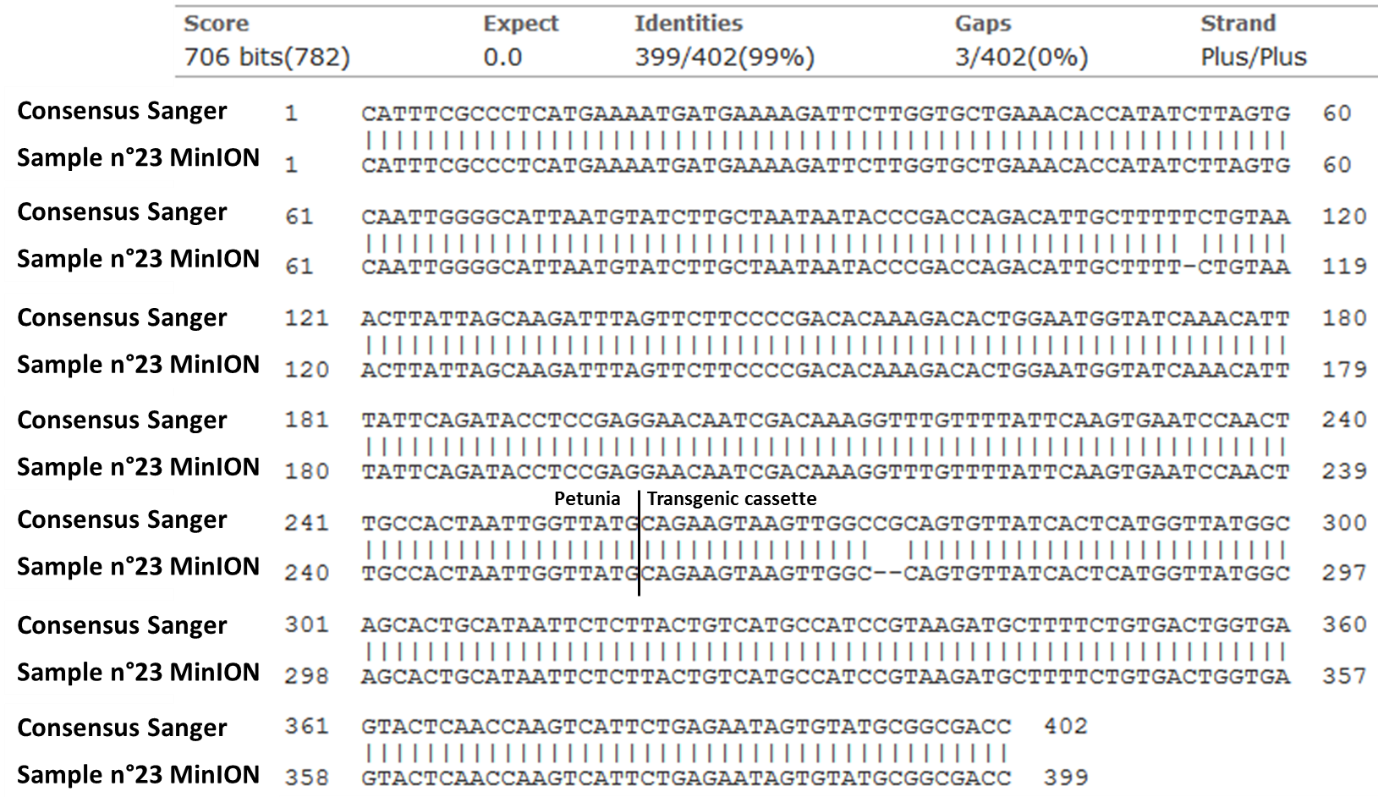 |

**Supplementary file 7: Results of the blastx on NCBI with the identified petunia sequence from the transgene flanking region (Figure 2; Supplementary file 6).**

| **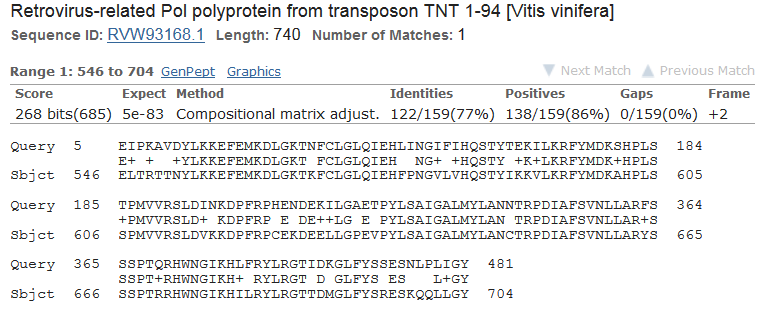** |
| --- |

**Supplementary file 8: List of used barcodes associated to the petunia samples.** The samples 1 to 11 were pooled for the first sequencing run while the samples 12 to 18 and 21 to 23 were pooled for the second sequencing run. The barcode sequences are described in the protocol of the EXP-NBD103 kit from Oxford Nanopore Technologies.

| **Samples** | **Barcode number** | **Barcode sequence** |
| --- | --- | --- |
| 1 | Barcode 2 | TCGATTCCGTTTGTAGTCGTCTGT |
| 2 | Barcode 3 | GAGTCTTGTGTCCCAGTTACCAGG |
| 3 | Barcode 4 | TTCGGATTCTATCGTGTTTCCCTA |
| 4 | Barcode 5 | CTTGTCCAGGGTTTGTGTAACCTT |
| 5 | Barcode 6 | TTCTCGCAAAGGCAGAAAGTAGTC |
| 6 | Barcode 7 | GTGTTACCGTGGGAATGAATCCTT |
| 7 | Barcode 8 | TTCAGGGAACAAACCAAGTTACGT |
| 8 | Barcode 9 | AACTAGGCACAGCGAGTCTTGGTT |
| 9 | Barcode 10 | AAGCGTTGAAACCTTTGTCCTCTC |
| 10 | Barcode 11 | GTTTCATCTATCGGAGGGAATGGA |
| 11 | Barcode 12 | CAGGTAGAAAGAAGCAGAATCGGA |
| 12 | Barcode 4 | TTCGGATTCTATCGTGTTTCCCTA |
| 13 | Barcode 5 | CTTGTCCAGGGTTTGTGTAACCTT |
| 14 | Barcode 6 | TTCTCGCAAAGGCAGAAAGTAGTC |
| 15 | Barcode 7 | GTGTTACCGTGGGAATGAATCCTT |
| 16 | Barcode 8 | TTCAGGGAACAAACCAAGTTACGT |
| 17 | Barcode 9 | AACTAGGCACAGCGAGTCTTGGTT |
| 18 | Barcode 10 | AAGCGTTGAAACCTTTGTCCTCTC |
| 21 | Barcode 1 | AAGAAAGTTGTCGGTGTCTTTGTG |
| 22 | Barcode 2 | TCGATTCCGTTTGTAGTCGTCTGT |
| 23 | Barcode 3 | GAGTCTTGTGTCCCAGTTACCAGG |
